# Shifting mutational constraints in the SARS-CoV-2 receptor-binding domain during viral evolution

**DOI:** 10.1126/science.abo7896

**Published:** 2022-06-28

**Authors:** Tyler N. Starr, Allison J. Greaney, William W. Hannon, Andrea N. Loes, Kevin Hauser, Josh R. Dillen, Elena Ferri, Ariana Ghez Farrell, Bernadeta Dadonaite, Matthew McCallum, Kenneth A. Matreyek, Davide Corti, David Veesler, Gyorgy Snell, Jesse D. Bloom

**Affiliations:** ^1^ Basic Sciences Division, Fred Hutchinson Cancer Research Center, Seattle, WA 98109, USA.; ^2^ Department of Genome Sciences, University of Washington, Seattle, WA 98109, USA.; ^3^ Medical Scientist Training Program, University of Washington, Seattle, WA 98109, USA.; ^4^ Molecular and Cellular Biology Graduate Program, University of Washington, Seattle, WA 98109, USA.; ^5^ Howard Hughes Medical Institute, Seattle, WA 98109, USA.; ^6^ Vir Biotechnology, San Francisco, CA 94158, USA.; ^7^ Department of Biochemistry, University of Washington, Seattle, WA 98195, USA.; ^8^ Department of Pathology, Case Western Reserve University School of Medicine, Cleveland, OH 44106, USA.; ^9^ Humabs BioMed SA, a subsidiary of Vir Biotechnology, 6500 Bellinzona, Switzerland.

## Abstract

SARS-CoV-2 has evolved variants with substitutions in the spike receptor-binding domain (RBD) that impact its affinity for ACE2 receptor and recognition by antibodies. These substitutions could also shape future evolution by modulating the effects of mutations at other sites—a phenomenon called epistasis. To investigate this possibility, we performed deep mutational scans to measure the effects on ACE2 binding of all single amino-acid mutations in the Wuhan-Hu-1, Alpha, Beta, Delta, and Eta variant RBDs. Some substitutions, most prominently N501Y, cause epistatic shifts in the effects of mutations at other sites. These epistatic shifts shape subsequent evolutionary change, for example enabling many of the antibody-escape substitutions in the Omicron RBD. These epistatic shifts occur despite high conservation of the overall RBD structure. Our data shed light on RBD sequence-function relationships and facilitate interpretation of ongoing SARS-CoV-2 evolution.

The SARS-CoV-2 spike receptor-binding domain (RBD) has evolved rapidly since the virus emerged (*1*). We previously used deep mutational scanning to experimentally measure the impact of all single amino-acid mutations on the ACE2-binding affinity of the ancestral Wuhan-Hu-1 RBD (*2*). These measurements have helped inform surveillance of SARS-CoV-2 evolution. For example, we identified the N501Y mutation as enhancing ACE2-binding affinity prior to the emergence of this consequential mutation in the Alpha variant (*3*).

However, as proteins evolve, the impacts of individual amino acid mutations can shift, a phenomenon known as epistasis (*4*). For example, the same N501Y mutation that enhances SARS-CoV-2 binding to ACE2 severely impairs ACE2 binding by SARS-CoV-1 and other divergent sarbecoviruses (*5*). Furthermore, N501Y epistatically enabled other affinity-enhancing mutations that emerged in the Omicron variant of SARS-CoV-2 (*6*–*8*). To more systematically understand how epistasis shifts the effects of mutations, we performed deep mutational scans to measure the impacts of all individual amino-acid mutations in SARS-CoV-2 variant RBDs.

We constructed comprehensive site-saturation mutagenesis libraries in the ancestral Wuhan-Hu-1 RBD (201 residues) and RBDs from four variants: Alpha (N501Y), Beta (K417N+E484K+N501Y), Delta (L452R+T478K) and Eta (E484K). We cloned these mutant libraries into a yeast-surface display platform and determined the impact of every amino acid mutation on ACE2-binding affinity and yeast surface-expression levels by FACS and high-throughput sequencing (figs. S1 and S2 and data S1) (2). The effect of each mutation on ACE2 binding is shown in Fig. 1, and an interactive version of this figure is available at https://jbloomlab.github.io/SARS-CoV-2-RBD_DMS_variants/RBD-heatmaps. We used monomeric ACE2 ectodomain to measure 1:1 binding affinities, which provide more granularity to reveal affinity-enhancing effects compared to our previous measurements using the natively dimeric ACE2 ligand, where some mutational effects are masked by avidity (fig. S1F) (2). Notably, mutant effects on ACE2 binding and protein expression in yeast-displayed RBD have been shown to closely correlate with ACE2 binding and protein expression in the context of full spike trimers displayed on mammalian cells (*9*, *10*).

**Fig. 1.**
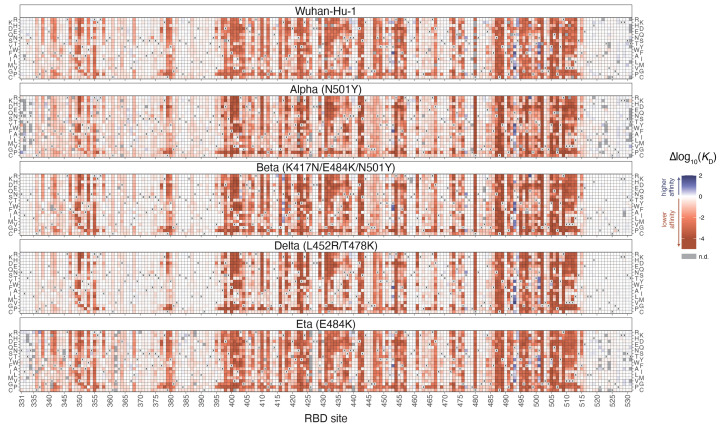
Deep mutational scanning maps of ACE2-binding affinity for all single amino acid mutations in five SARS-CoV-2 RBD variants. The impact on ACE2 receptor-binding affinity (Δlog_10_(*K*_D_)) of every single amino-acid mutation in SARS-CoV-2 RBDs, as determined by high-throughput titration assays (fig. S1). The wildtype amino acid in each variant is indicated with an “x”, and gray squares indicate missing mutations in each library. An interactive version of this map is at https://jbloomlab.github.io/SARS-CoV-2-RBD_DMS_variants/RBD-heatmaps, and raw data are in data S1. The effects of mutations on RBD surface expression are in fig. S2.

We identified sites where the impacts of mutations differ between RBD variants (Fig. 2 and figs. S3 and S4), reflecting epistasis among the substitutions that distinguish SARS-CoV-2 variants and other mutations across the RBD. These epistatic shifts in mutational effects on ACE2 binding are primarily attributable to the N501Y mutation: the effects of mutations in the Delta (L452R+T478K) and Eta (E484K) RBDs are similar to those in the ancestral Wuhan-Hu-1 RBD, and the differences in the Beta (K417N+E484K+N501Y) RBD largely recapitulate those in the Alpha RBD containing N501Y alone (Fig. 2, A and B). One exception is a unique epistatic shift in the effects of mutations to serine or threonine at site 419 in the Beta RBD that introduce an N-linked glycosylation motif when an asparagine is present via the K417N mutation (fig. S3D).

**Fig. 2.**
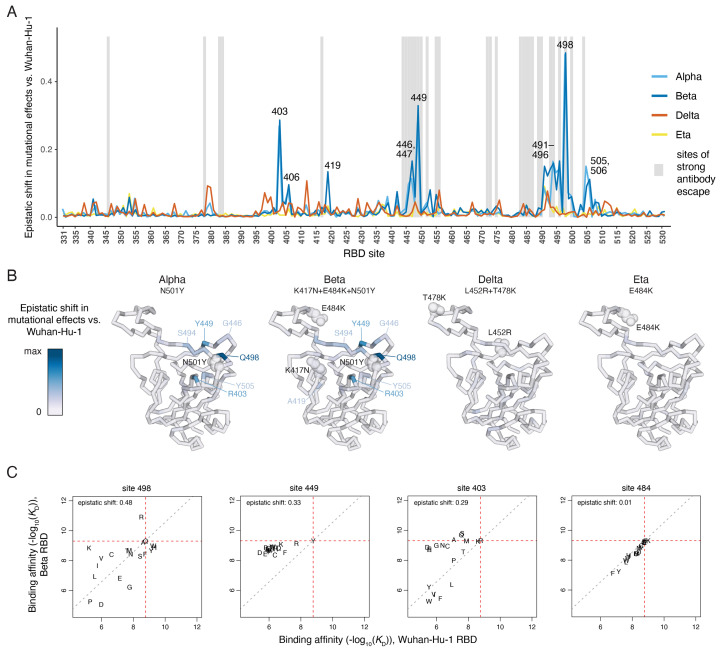
Epistatic shifts in mutational effects across RBD variants. (**A**) The shift in mutational effects on ACE2 binding at each RBD site between the indicated variant and Wuhan-Hu-1. An interactive version of this plot is at https://jbloomlab.github.io/SARS-CoV-2-RBD_DMS_variants/epistatic-shifts. The epistatic shift is calculated as the Jensen-Shannon divergence in the set of Boltzmann-weighted affinities for all amino acids at each site. Gray shading indicates sites of strong antibody escape based on prior deep mutational scanning of the Wuhan-Hu-1 RBD (*11*). (**B**) Ribbon diagram of the Wuhan-Hu-1 RBD structure (PDB 6M0J) colored according to epistatic shifts. Labeled spheres indicate residues that are mutated in each RBD variant. (**C**) Mutation-level plots of epistatic shifts at sites of interest. Each scatter plot shows the measured affinity of all 20 amino acids in the Beta versus Wuhan-Hu-1 RBD. Red dashed lines mark the parental RBD affinities, and the gray dashed line indicates the additive (non-epistatic) expectation. Epistatic shifts can reflect idiosyncratic mutation-specific shifts (e.g., site 498) or global changes in mutational sensitivity at a site (e.g., site 449). Site 484 does not have a substantial epistatic shift and is shown for comparison. See fig. S3 for scatterplots of additional sites of interest. See fig. S4 for epistatic shifts in mutational effects on RBD expression.

The RBD sites that exhibit notable epistatic shifts due to N501Y fall into three structural groups (Fig. 2B). The largest shift in mutational effects is at the direct N501-contact residue Q498 (Fig. 2C), together with further epistatic shifts at sites 491-496 comprising the central beta strand of the ACE2-contact surface (Fig. 2B and fig. S3A). A second cluster of sites exhibiting epistatic shifts in the presence of N501Y include 446, 447, and 449, which do not directly contact N501 but are spatially adjacent to residue 498 (Fig. 2, B and C, and fig. S3B). A third group of sites that epistatically shift due to N501Y includes residue R403 (Fig. 2C), together with several residues (505, 506, and 406) that structurally link site 501 to site 403 (fig. S3C).

Some of these epistatic shifts are of clear relevance during the evolution of SARS-CoV-2. One of the strongest epistatic shifts is the potentiation of Q498R by N501Y (Figs. 2C and 3A). Although Q498R alone weakly reduces ACE2 affinity in the Wuhan-Hu-1 RBD, it confers a 25-fold enhancement in affinity when present in conjunction with N501Y (which itself improves binding 15-fold in Wuhan-Hu-1), such that the double mutant has a 387-fold increased binding affinity. The Q498R/N501Y double mutation was first discovered in directed evolution studies (*6*) and is present in the RBD of the Omicron BA.1 and BA.2 variants (*8*). The epistasis between these two mutations is crucial for enabling the Omicron RBD to bind ACE2 with high affinity despite having a large number of mutations (*12*–*14*). Specifically, the set of mutations in the Omicron RBD are predicted to strongly impair ACE2 affinity based on their summed single-mutant effects in Wuhan-Hu-1 (Fig. 3B, left), but their summed single-mutant effects in the Beta background (which has N501Y) is about zero (Fig. 3B, right), consistent with the actual affinity of the Omicron RBD for ACE2. Therefore, the affinity buffer conferred by the epistatic Q498R/N501Y pair enables the Omicron spike to tolerate other mutations that decrease ACE2 binding (Fig. 3B and fig. S5A) but contribute to antibody escape (fig. S5, B and C) (*11*). Consistent with these affinity measurements, introducing R498Q and Y501N reversions into the Omicron BA.1 spike reduces cell entry by spike-pseudotyped lentiviral particles, suggesting that the remaining Omicron RBD mutations are deleterious without buffering by Q498R/N501Y (Fig. 3C and fig. S6, A and B).

**Fig. 3.**
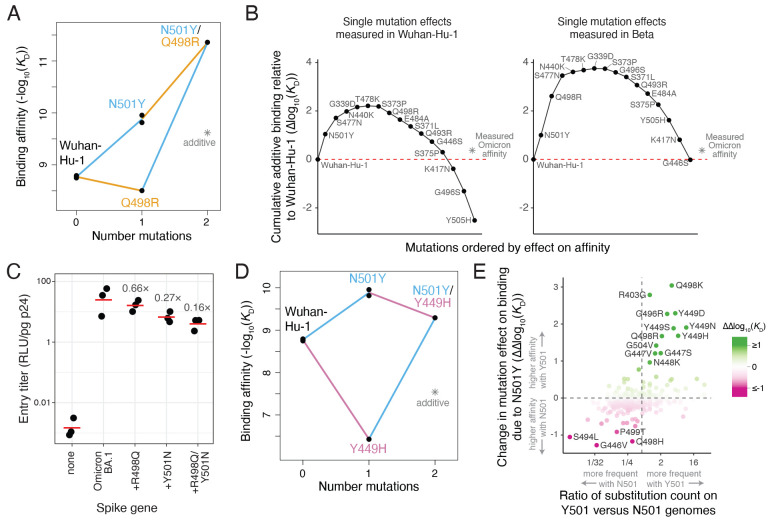
Functional and evolutionary relevance of epistatic interactions. (**A**) Double mutant cycle diagram illustrating the positive epistasis interaction between N501Y and Q498R. Asterisk indicates expected double-mutant binding affinity assuming additivity. (**B**) Affinity-buffering of Omicron BA.1 mutations. Each diagram shows the cumulative addition of individually measured effects on ACE2-binding affinity (Δlog_10_(*K*_D_)) for each single RBD substitution in Omicron BA.1 as measured in the Wuhan-Hu-1 (left) or Beta (right) RBDs. Mutation effect is calculated in the labeled direction even when the reference state in a background differs, e.g., N501Y in the Beta background is the opposite-sign effect of the measured Y501N mutation. Red line marks the Wuhan-Hu-1 affinity, and asterisk the actual affinity of the Omicron BA.1 RBD relative to Wuhan-Hu-1 as measured in (*12*). See also fig. S5, A to C. (**C**) Efficiency of entry of Omicron BA.1 (or reversion mutant) spike-pseudotyped lentivirus on a HEK-293T cell line expressing low levels of ACE2 (fig. S6, A and B). Labels indicate fold-decrease in geometric mean (red bar) of biological triplicate measurements. (**D**) Double mutant cycle illustrating positive epistasis between N501Y and Y449H. (**E**) Impact of epistasis on SARS-CoV-2 sequence evolution. Plot illustrates the change in a mutation’s effect between Alpha (N501Y) versus Wuhan-Hu-1 deep mutational scanning data, versus the ratio in number of observed occurrences of the substitution in genomes containing N501 versus Y501 in a global SARS-CoV-2 phylogeny as of 25 May, 2022 (*22*). Note that we are counting substitution occurrence as an event on the phylogeny independent of the number of offspring of a node, and not the raw number of sequenced genomes with which a mutation is observed. A pseudocount was added to all substitution counts to enable ratio comparisons, and substitutions that were observed <2 times in total are excluded. Color scale reinforces the ΔΔlog_10_(*K*_D_) metric on the y-axis. Labeled mutations are those with |ΔΔlog_10_(*K*_D_)| > 0.9. Vertical line at *x* ~ 0.6 marks equal relative occurrence on Y501 versus N501 genomes given the larger number of substitutions that had been observed on N501 genomes.

There is also evolutionary relevance of the epistasis of N501Y with mutations on the 446-449 loop, which comprises the epitope for an important class of human antibodies (*15*, *16*). Although mutations to G446 escape this class of antibodies in the Wuhan-Hu-1 RBD (*16*, *17*), these mutations incur stronger ACE2-binding deficits in the N501Y background (figs. S3B and S5D). Conversely, mutations to Y449 strongly decrease ACE2-binding affinity in the Wuhan-Hu-1 RBD but are better tolerated when accompanied by N501Y (Figs. 2C and 3D). Mutations to Y449 can escape monoclonal antibodies (fig. S6, C to E) (*15*, *18*) and reduce neutralization by polyclonal sera (*19*, *20*), and have been described in several variants that also contain N501Y including the C.1.2, A.29, and B.1.640 lineages (*19*, *21*).

To more systematically examine how epistatic shifts caused by N501Y impact patterns of sequence variation during SARS-CoV-2 evolution, we counted the occurrence of substitutions on a global SARS-CoV-2 phylogeny (*22*). Substitutions more often occurred in backgrounds containing the amino acid at site 501 with which they had more favorable epistasis with respect to ACE2 affinity (Fig. 3E). Therefore, epistatic shifts caused by N501Y have directly impacted patterns of mutation accumulation in prior SARS-CoV-2 evolution, and our data enable identification of mutations like those at site Y449 whose evolutionary relevance may grow if N501Y variants continue to predominate. Of note, Q498R had not previously occurred disproportionately on Y501 genomes until its predominance in Omicron lineages. We hypothesize that the strong affinity gain caused by the Q498R/N501Y double mutant (Fig. 3A) is not directly advantageous itself, but rather becomes beneficial in Omicron because it can buffer other beneficial antibody-escape mutations as described above.

Other common combinations of mutations are not involved in specific epistatic interactions. For instance, substitutions at sites 417, 484, and 501 arose together in the Beta and Gamma variants. Early studies disagree on whether there is epistasis among these mutations with respect to ACE2 binding (*6*, *23*, *24*), but our data demonstrate strict additivity (figs. S3E and S5E). The co-occurrence of mutations at these three sites in SARS-CoV-2 variants may instead reflect antigenic selection for E484 and K417 mutants (which escape different classes of neutralizing antibodies (*15*)), while N501Y might globally compensate for the affinity-decreasing effect of K417 mutations. These examples illustrate how N501Y can enable viral evolution through specific epistatic modulation (e.g., Y449 mutations) as well as non-specific affinity-buffering (e.g., K417N).

To examine the structural basis for epistatic shifts in mutational effects, we examined ACE2-bound RBD crystal structures of the Wuhan-Hu-1 and Beta RBDs (*25*, *26*), including a newly determined crystal structure of the ACE2-bound Beta RBD (plus antibodies S304 and S309) at 2.45Å resolution (table S1). These comparisons do not reveal clear structural perturbations that explain epistatic shifts between the Wuhan-Hu-1 and Beta RBDs: residues with large epistatic shifts between backgrounds show similar extents of variation between Wuhan-Hu-1 and Beta structures as they show within replicate structures of Wuhan-Hu-1 or Beta itself (fig. S7). More broadly, there is minimal change between Wuhan-Hu-1 and Beta RBD backbones (Fig. 4A and fig. S8A), and we did not detect any correlation between structural displacement of backbone or sidechain atoms in variant RBD structures and epistatic shifts in mutational effects (Fig. 4B and fig. S8, B to E). These observations indicate that epistatic shifts in mutant effects occur despite conservation of the global static RBD structure.

**Fig. 4.**
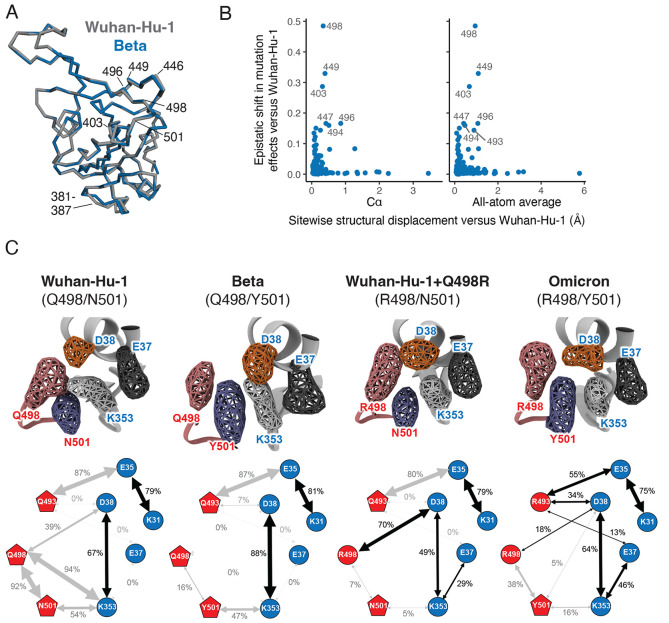
Epistatic shifts are not accompanied by large structural perturbations. (**A**) Global alignment of the Wuhan-Hu-1 (PDB 6M0J) and Beta (PDB 7EKG) RBD backbones. Key sites are labeled. (**B**) Correlation between the extent of epistatic shift in mutational effects at a site and its structural perturbation in Beta versus Wuhan-Hu-1 RBDs (backbone Cα or all-atom average displacement from aligned X-ray crystal structures). See figs. S7 and S8 for additional details. (**C**) Molecular dynamics simulation of RBD variants bound to ACE2. Volumetric maps (top) show the 3D space occupied by key residues over the course of simulation. Cartoon diagrams (bottom) illustrate the fraction of simulation frames in which a salt bridge (black arrow) or polar or nonpolar (gray arrow) contact is formed between residue pairs (fig. S9C). See fig. S9A for equivalent diagrams for Omicron+Y501N (R498/N501 for comparison with Wuhan-Hu-1+Q498R) and fig. S9B for apo ACE2. See fig. S9C for histograms of contact distances over the course of the simulations.

To explore the cause of epistasis between Q498R and N501Y (Fig. 3A), we performed molecular dynamics simulations of the Wuhan-Hu-1 (Q498/N501), Beta (Q498/Y501) and Omicron (R498/Y501) RBDs bound to ACE2 (*14*, *25*), in addition to in silico mutated complexes of Wuhan-Hu-1+Q498R and Omicron+Y501N (Fig. 4C and fig. S9). The Wuhan-Hu-1 structure features a stable polar contact network between ACE2 residues D38 and K353 and RBD residue Q498. The affinity-enhancing N501Y substitution present in Beta repositions K353_ACE2_ in an orientation that reinforces the D38_ACE2_ salt bridge but disrupts all Q498 contacts. In contrast, the affinity-decreasing Q498R mutation alone improves the coordination between residue 498 and D38_ACE2_ but leaves K353_ACE2_ incompletely satisfied. In Omicron, the Q498R and N501Y combination pose K353_ACE2_ in a stable rotamer that maintains the D38_ACE2_ salt bridge and reanimates the E37_ACE2_ salt bridge present in the *apo* ACE2 structure (fig. S9B) while adding a new minor salt bridge contact between R498 and D38_ACE2_. This complex epistatic reconfiguration of a polar contact network illustrates how the dynamic basis of RBD:ACE2 interaction leads to dynamic evolutionary variability.

Overall, SARS-CoV-2 has explored a diverse set of mutations during its evolution in humans. Our results show how this ongoing evolution is itself shaping potential future routes of change by shifting the effects of key mutations on receptor-binding affinity. Other human coronaviruses have proven adept at escaping from antibody immunity (*27*) because they can undergo extensive evolutionary remodeling of the amino-acid sequence of their receptor-binding domain while retaining high receptor affinity (*28*, *29*). Our work provides large-scale sequence-function maps that help understand how a similar process may play out for SARS-CoV-2.

## Supplementary Materials

### This PDF file includes:

Materials and Methods Fig. S1 to S9 Table S1 References (*31**–**62*)

### Other Supplementary Material for this manuscript includes the following:

MDAR Reproducibility Checklist
