# Supplementary material for: Shifting mutational constraints in the SARS-CoV-2 receptor-binding domain during viral evolution
Source: Science. 2022 Jun 28:eabo7896. doi: 10.1126/science.abo7896 (PMC9273037; doi:10.1126/science.abo7896)
Supplement: Supplementary file 1 — Materials and Methods Fig. S1 to S9 Table S1 References (31–62) [file science.abo7896_sm.pdf]

Science
AAAS

Supplementary Materials for

Shifting mutational constraints in the SARS-CoV-2 receptor-binding domain during viral evolution

Tyler N. Starr *et al.*

Corresponding authors: Tyler N. Starr, tstarr@fredhutch.org; Jesse D. Bloom, jbloom@fredhutch.org

DOI: 10.1126/science.abo7896

The PDF file includes:

Materials and Methods
Fig. S1 to S9
Table S1
References

Other Supplementary Material for this manuscript includes the following:

MDAR Reproducibility Checklist
Data S1

Materials and Methods

Library generation

Site-saturation mutagenesis libraries spanning all 201 positions in variant RBDs were created as described for the Beta library by Greaney et al. (*16*). Briefly, a plasmid encoding the Wuhan-Hu-1 RBD (Genbank MN908947, residues N331–T531) was mutagenized to create variant RBDs Alpha (N501Y), Beta (K417N+E484K+N501Y, described in (*16*)), Delta (L452R+T478K), and Eta (E484K). Site saturation mutant libraries in each variant background were produced by Twist Bioscience, introducing precise codon mutations to encode the 20 possible amino acids with no stop codons at each RBD position. Libraries were provided as dsDNA oligos, to which we appended N16 barcodes via PCR primer addition. Failed positions in the Twist mutagenesis were mutagenized in-house using NNS degenerate primers and pooled with the respective Twist libraries for N16 barcoding. Barcoded libraries were cloned into a recipient vector backbone for yeast-surface display (2) via Gibson Assembly. Libraries were electroporated into *E. coli* (NEB C3020K) and plated in duplicate at a target bottleneck of 50,000 unique barcodes per library (100,000 for Delta libraries, which were processed subsequent to the other libraries). Colonies were scraped from transformations of each RBD variant library, and plasmid was purified and transformed into the AWY101 *Saccharomyces cerevisiae* yeast strain (*31*, gift from Eric Shusta).

As described (2, *16*), plasmid samples were sequenced using a PacBio Sequel IIe to generate long sequence reads that span the N16 barcode and RBD coding sequence. Raw CCS reads are available on the NCBI Sequence Read Archive, BioProject PRJNA770094, BioSample SAMN25941479 (together with prior Beta PacBio reads under BioSample SAMN22208699 (*16*)). Reads were processed using alignparse (version 0.2.4) (*32*) to generate a table linking each N16 barcode to its unique RBD mutant. Barcode lookup tables for each variant background are available at https://github.com/jbloomlab/SARS-CoV-2-RBD_DMS_variants/tree/main/results/variants and https://github.com/jbloomlab/SARS-CoV-2-RBD_Delta/blob/main/results/variants/codon_variant_table.csv.

ACE2-binding titration deep mutational scanning experiments

The ACE2-binding affinity of each RBD mutant was determined via massively parallel yeast-surface display titrations (2, *33*). Titrations were performed on pooled mutant libraries of the Wuhan-Hu-1, Alpha, Beta, and Eta variants, while the Delta libraries were assayed independently. For the Wuhan-Hu-1, Alpha, Beta, and Eta pools, two experimental replicate titrations were performed on the pooled "replicate 1" libraries, and a single titration was performed on the pooled "replicate 2" libraries. For the Delta library, a single experimental replicate was performed with each library replicate. Frozen yeast libraries were thawed, grown overnight in SD-CAA media (6.7 g/L Yeast Nitrogen Base, 5.0 g/L Casamino acids, 2.13 g/L MES, and 2% w/v dextrose), and backdiluted to 0.67 OD600 in SG-CAA+0.1%D (SD-CAA with 2% galactose + 0.1% dextrose replacing the 2% dextrose) to induce RBD surface expression, which proceeded for 16-18 hours at room temperature with mild agitation.

Induced cells were washed with PBS-BSA (0.2 mg/L), and split into incubations with biotinylated monomeric human ACE2 protein (ACROBiosystems AC2-H82E8) across a concentration range from 10^{-6} to 10^{-13} M at 1-log intervals, plus a 0 M ACE2 sample. Incubations equilibrated overnight at room temperature with mixing. Yeast were washed with ice-cold PBS-BSA and fluorescently labeled with 1:100 FITC-conjugated chicken anti-Myc (Immunology

Consultants CMYC-45F) to detect yeast-displayed RBD protein and 1:200 PE-conjugated streptavidin (Thermo Fisher S866) to detect bound ACE2. At each ACE2 sample concentration, single RBD^+ cells were partitioned into bins of ACE2 binding (PE fluorescence) using a BD FACS Aria II as shown in Fig. S1A. A minimum of 10 million cells were collected at each sample concentration. Cells in each bin were grown overnight in 1 mL SD-CAA+pen-strep, and plasmid was isolated using 96-well yeast miniprep kits (Zymo D2005) according to manufacturer instructions, with the addition of an extended (>2 hr) Zymolyase treatment and a -80°C freeze/thaw cycle prior to cell lysis. N16 barcodes in each post-sort sample were PCR amplified as described in Starr et al. (2) and submitted for Illumina HiSeq 50bp single end sequencing. Sequencing reads are available on the NCBI Sequence Read Archive, BioProject PRJNA770094, BioSample SAMN25944367.

Demultiplexed Illumina barcode reads were aligned to library barcodes in barcode-mutant lookup tables using dms_variants (version 0.8.9), yielding a table of counts of each barcode in each FACS bin which is available at https://github.com/jbloomlab/SARS-CoV-2-RBD_DMS_variants/blob/main/results/counts/variant_counts.csv. Read counts in each FACS bin were downweighted by the ratio of total sequence reads from a bin to the number of cells that were sorted into that bin from the FACS log.

We estimated the level of ACE2 binding of each barcoded mutant at each ACE2 sample concentration based on its distribution of counts across FACS bins as the simple mean bin (*34*) as described in (2). We determined the binding constant K_D describing the affinity of each barcoded mutant for ACE2 along with free parameters *a* (titration response range) and *b* (titration curve baseline) via nonlinear least-squares regression using the standard non-cooperative Hill equation relating the mean sort bin to the ACE2 labeling concentration:

$$\text{bin} = a \times [\text{ACE2}] / ([\text{ACE2}] + K_D) + b$$

The measured mean bin value at a given ACE2 concentration was excluded from curve fitting for a barcode if fewer than 2 counts were observed across the four FACS bins at that ACE2 concentration. Individual concentration points were also excluded from the curve fit if they exhibited bimodality (>40% of counts of a barcode were found in two non-consecutive bins). To avoid errant fits, we constrained the baseline fit parameter *b* to be between 1 and 1.5, the response parameter *a* between 2 and 3, and the K_D parameter between 1e-15 and 1e-5. The fit for a barcoded mutant was discarded if the average count across all sample concentrations was below 2, or if more than one sample concentration was missing or excluded. We also discarded curve fits where the normalized mean square residual (residuals normalized relative to the fit response parameter *a*) was >40 times the median normalized mean square residual across all titration fits. Final K_D binding constants were expressed as $-\log_{10}(K_D)$, where higher values indicate higher binding affinity. The computational pipeline for computing per-barcode binding constants is available at https://github.com/jbloomlab/SARS-CoV-2-RBD_DMS_variants/blob/main/results/summary/compute_binding_Kd.md.

Because most mutants in the library were independently associated with more than one N16 barcode, we were able to average across internal replicates to derive final mutant affinities. Barcode affinities associated with identical RBD genotypes were first averaged within each experimental replicate. The correlations in per-replicate collapsed affinities are shown in Fig. S1C. The final average was then determined as the average of each replicate value. The final collapsed K_D for each mutant is given in Data S1 and https://github.com/jbloomlab/SARS-CoV-2-RBD_DMS_variants/blob/main/results/final_variant_scores/final_variant_scores.csv. The median mutant's ACE2 binding affinity measurement collapses 15 total barcodes across the

replicate titration experiments (Fig. S1E). The number of independent barcodes collapsed into each mutation's final K_D is reported in the raw data table linked above.

RBD expression deep mutational scanning experiments

Libraries were grown and induced for RBD expression as described above. Induced cells were washed, labeled with 1:100 FITC-conjugated chicken anti-Myc, and washed in preparation for FACS. Single cells were partitioned into bins of RBD expression (FITC fluorescence) using a BD FACS Aria II as shown in Fig. S1B. A total of >10 million viable cells (estimated by plating of post-sort dilutions) were collected for each library. Cells in each bin were grown out, plasmid isolated, and N16 barcodes sequenced as described above, except read count downweighting used the post-sort colony counts instead of the FACS log counts. Sequencing reads are available on the NCBI Sequence Read Archive, BioProject PRJNA770094, BioSample SAMN25944367.

We estimated the level of RBD expression (mean fluorescence intensity, MFI) of each barcoded mutant based on its distribution of counts across FACS bins and the known log-transformed fluorescence boundaries of each sort bin using a maximum likelihood approach (*2*, *34*), implemented using the fitdistrplus package (version 1.0.14) in R (*35*). Expression measurements were discarded for barcodes for which fewer than 10 counts were observed across the four FACS bins. The full pipeline for computing per-barcode expression values is available at https://github.com/jbloomlab/SARS-CoV-2-RBD_DMS_variants/blob/main/results/summary/compute_expression_meanF.md. Final mutant expression values were collapsed within and across replicates as described above (correlation between replicates shown in Fig. S1D), with a median of 10 barcodes collapsed into each mutant expression measurement (Fig. S1E). Final mutant expression values are available in Data S1 and https://github.com/jbloomlab/SARS-CoV-2-RBD_DMS_variants/blob/main/results/final_variant_scores/final_variant_scores.csv.

Quantification of epistasis

Epistatic shifts at each site between pairs of RBD variants were quantified via a summary metric, Jensen-Shannon divergence, that captures variation in the 20 amino-acid-level affinity or expression phenotypes measured at a position (*36*). First, affinity (dissociation constant K_D) or expression (MFI) phenotypes f_i of each mutant i at a site were transformed to a probability analog π_i:

$$\pi_i = e^{-ln(f_i)} / \sum_i e^{-ln(f_i)}$$

Note that for affinity, the resulting π_i values correspond to Boltzmann weights, since -ln(K_D) is proportional to the free energy in units of $k_B T$. The Jensen-Shannon divergence between vectors p and q representing the 20 amino acid π_i at a site in two RBD variants is the average Kullback-Leibler divergence of p and q from their per-index-mean vector m:

$$JSD(p||q) = \frac{D(p\,||\,m) + D(q\,||\,m)}{2}$$

where the Kullback-Leibler divergence is calculated as:

$$D(p\,||\,m) = \sum_i p(i) log(\frac{p(i)}{m(i)})$$

The Jensen-Shannon divergence ranges from 0 for two vectors of probabilities that are identical to 1 for two vectors that are completely dissimilar. For context, scatterplots in Fig. 2C and Figs. S3, S4C illustrate the amino-acid-level epistatic perturbations that give rise to a range of Jensen-Shannon divergence values. To avoid noisier measurements artifactually inflating the epistatic shift metric, a given amino acid mutation was only included in the comparison between a pair of RBD variants if its final measurement was averaged across 3 or more individual barcodes in each RBD background. The calculation of epistatic shifts can be found at https://github.com/jbloomlab/SARS-CoV-2-RBD_DMS_variants/blob/main/results/summary/epistatic_shifts.md.

For epistatic interactions between particular mutations (e.g., Fig. 3A,B,D and Fig. S5D,E), the expected difference in binding of a multiply mutated genotype compared to wildtype in the absence of epistasis is the sum of the $\Delta\log_{10}(K_D)$ measurements of the component single mutations. Any deviation from this addition is a reflection of epistasis. An observed phenotype that is higher than the expected additive phenotype is considered "positive" epistasis, while an observed phenotype that is lower than the additive expectation is considered "negative" epistasis.

Analysis of SARS-CoV-2 genetic variation

A real-time phylogenetic tree of global SARS-CoV-2 genomes was used to count substitution accrual on internal branches of the SARS-CoV-2 phylogeny. The SARS-CoV-2 mutation-annotated tree described by McBroome et al. (*22*) was downloaded on May 25, 2022. Nucleotide mutations annotated on the tree were converted to amino acid mutations using matUtils (version 0.4.8) (*22*), using the Wuhan-Hu-1 genome (NCBI RefSeq NC_045512.2) as a reference. Amino acid substitutions were tabulated, excluding substitutions that occurred to isolated terminal branches. Substitutions were counted as occurring on N501 versus Y501 genomes, and substitutions that occurred coincidental with a 501 substitution were counted as accruing on the derived 501 state from that branch. A pseudocount of 1 was added to all substitution counts to enable log-ratio comparison of mutation accrual on N501 versus Y501 genomes (Fig. 3E). Note that we are counting occurrence of substitutions as individual ancestral events on the SARS-CoV-2 phylogeny, and not mutation frequencies which are conflated by variation in amplification of certain lineages and biases in depth of sampling over space and time.

Pseudovirus generation, titering, and neutralization experiments

A spike-pseudotyped lentiviral platform (*37*) was used to measure entry efficiency and antibody neutralization of spike variants. Single and double amino acid mutations and the entire suite of mutations found in Omicron BA.1 (*8*) were introduced into a previously described spike-expression plasmid containing D614G and a 21-amino-acid deletion in the cytoplasmic tail (Addgene 158762) (*37*). Plasmids were purified in triplicate for independent viral rescues from separate plasmid stocks.

Spike pseudotyped lentiviral particles were produced in human HEK-293T cells (ATCC CRL-3216). 5e5 cells per well were seeded in 6-well plates in 2mL D10 growth media (Dulbecco's Modified Eagle Medium with 10% heat-inactivated fetal bovine serum, 2 mM L-glutamine, 100 U/mL penicillin, and 100 µg/mL streptomycin) at 37°C in a humidified 5% CO_2 incubator. 24 hours later, cells were transfected using BioT (Bioland Scientific) with 340 ng of spike expression plasmid (or no spike control), 1,135 ng of a Luciferase_IRES_ZsGreen lentiviral backbone, and 865 ng of Gag/Pol lentiviral helper plasmid (BEI Resources NR-52517).

Media was changed 24 hours post-transfection. Approximately 65 hours post-transfection, viral supernatants were collected, filtered through a 0.45 μm syringe filter, and stored at -80°C. Particle concentration of each viral supernatant was determined in technical duplicate by p24 ELISA (Advanced Bioscience Laboratories Cat. # 5421) versus a known particle standard according to manufacturer instructions.

Entry titers of spike-pseudotyped lentiviral particles were determined on HEK-293T cell lines expressing high levels of human ACE2 (BEI Resources NR-52511) (*37*) and low levels of human ACE2. The low level ACE2 cells were created by genomically integrating single AttB_ACE2-miRFP670_IRES_mCherry-H2A-P2A-PuroR plasmids into HEK-293T LLP-Int-BFP-IRES-iCasp9-Blast Bxb1 landing pad cells (*37*), followed by selection with 1 μg/mL puromycin. Low ACE2 levels were achieved by using a "AATTTT[ATG]" Kozak sequence preceding ACE2 to decrease its steady-state abundance, unmasking entry deficits (*38*). All cells were grown in D10 media, and the ACE2-low cell line was supplemented with 1 μg/mL doxycycline to maintain ACE2 expression. To quantify ACE2 expression levels, HEK-293T cells expressing high, low or no ACE2 were resuspended in FACS buffer (PBS+2% BSA) and incubated for 1 h with 1:500 rabbit anti-ACE2 antibody (Abcam ab272500). Cells were washed with FACS buffer and labeled for 1 h with 1:3000 Alexa-Fluor-488 goat anti-rabbit IgG H&L (Abcam ab150077). Cells were washed with FACS buffer and fixed with 4% PFA. ACE2 expression (AF488 fluorescence) was measured via flow cytometry on a BD LSRFortesssa X50, and geometric mean fluorescence intensity was compared between samples (Fig. S6A).

For titering, ACE2-high and ACE2-low cells were seeded at 1.2e4 cells per well in poly-L-lysine-coated 96-well plates (Greiner 655930) in 50 uL D10 media (plus 1 μg/mL doxycycline for the ACE2-low cell line). 24 hours later, 100 μL of viral supernatants prepared across a 2-fold dilution series (1:2 to 1:256) were added to cells. Approximately 65 hours post-infection, luciferase activity was measured (Promega Bright-Glo, E2620). Relative luciferase unit (RLU) measures were averaged across viral dilutions within linear range and expressed relative to p24 particle concentration as the final p24-normalized entry titer (RLU/pg p24).

Antibody neutralization of spike-pseudotyped particles was measured on ACE2-high cell lines in technical duplicate. Cells were seeded in 96-well plates as described above for virus titering. 24 hours later, viral supernatants were diluted to a to a target of 400,000 RLU per well, and incubated for 1 hour at 37°C with monoclonal antibody across 8 four-fold dilution points starting at a concentration 400-fold higher than the expected IC50 (*18*). 100 μL of antibody-virus mixture was added to cells, and luciferase activity was measured ~65 hours post-infection as described for titering experiments. Fraction infectivity of each sample was calculated relative to a no-antibody well inoculated with the same viral supernatant in a matched row of the 96-well plate. We used neutcurve (https://jbloomlab.github.io/neutcurve, version 0.5.7) to calculate the inhibitory concentration 50% (IC50) of each antibody against each virus by fitting a Hill curve with fixed baseline 0 and plateau 1.

Recombinant protein production

SARS-CoV-2 Beta RBD for crystallization (residues 328-531 of S protein from GenBank NC_045512.2 with N-terminal signal peptide and C-terminal 8xHis-tag) was expressed in Expi293F (Thermo Fisher Scientific) cells in the presence of 10 μM kifunensine at 37°C and 8% CO2. Transfection was performed using the ExpiFectamine 293 Transfection Kit (Thermo Fisher Scientific). Cell culture supernatant was collected four days after transfection and supplemented with 10x PBS to a final concentration of 2.5x PBS (342.5 mM NaCl, 6.75 mM KCl and 29.75

mM phosphates). SARS-CoV-2 Beta RBD was purified using a 5 mL HisTalon Superflow cartridge (Takara Bio) followed by buffer exchange into PBS using a HiPrep 26/10 desalting column (Cytiva).

Human ACE2 for crystallization (residues 19-615 from Uniprot Q9BYF1 with a C-terminal thrombin cleavage site-TwinStrep-10xHis-GGG-tag, and N-terminal signal peptide) was expressed in Expi293F cells in the presence of 10 µM kifunensine at 37°C and 8% CO2. Transfection was performed using the Expi293 transfection kit (Thermo Fisher Scientific). Cell culture supernatant was collected five days after transfection and supplemented to a final concentration of 80 mM Tris-HCl pH 8.0, 100 mM NaCl, and then incubated with BioLock (IBA GmbH) solution. ACE2 was purified using a 1 mL StrepTrap HP column (Cytiva). Protein-containing fractions were pooled and digested with EndoH and thrombin at 4°C overnight. ACE2 was further purified by size exclusion chromatography using a Superdex 200 Increase 10/300 GL column (Cytiva) equilibrated in 20 mM Tris-HCl pH 7.5, 150 mM NaCl.

Crystallization, data collection, structure determination, and analysis

SARS-CoV-2 Beta RBD was mixed with a 1.4-fold molar excess hACE2, and a 1.3-fold molar excess of S304 Fab and S309 Fab. The complex was purified on a Superdex 200 10/300 GL column pre-equilibrated in 20 mM Tris-HCl pH 7.5, 150 mM NaCl. Crystals of the SARS-CoV-2 Beta RBD-hACE2-S304-S309 Fab complex were obtained at 20°C by sitting drop vapor diffusion. A total of 200 nL of the complex at 6 mg/mL was mixed with 200 nL mother liquor solution containing 10% w/v PEG 8000, 20% v/v ethylene glycol, 0.1 M Tris (base)/bicine pH 8.5, 3% w/v D-sorbitol. Crystals were flash frozen in liquid nitrogen.

Data were collected at Beamline 9-2 of the Stanford Synchrotron Radiation Lightsource facility in Stanford, CA and processed with the XDS software package (*39*) yielding a final dataset of 2.45 Å in space group P1. The SARS-CoV-2 Beta RBD-hACE2-S304-S309 complex structure was solved by molecular replacement using Phaser (*40*) from a starting model consisting of ACE2-RBD-S304-S309 (PDB: 7L0N). Several subsequent rounds of model building and refinement were performed using Coot (*41*), ISOLDE (*42*), Refmac5 (*43*), Phenix (*44*) and MOE (https://www.chemcomp.com), to arrive at a final model of the quaternary complex.

MD simulation

Coordinates of the Wuhan-Hu-1 RBD:hACE2 structure were prepared as previously described (*45*), where the RBD was taken from PDB 6M0J, hACE2 from PDB 1R42, and their complex generated by aligning the proteins to the CST 6M0J structure. Complex glycans were then added to the structure at positions 53, 90, 103, 322, 432, 546, and 690 on hACE2 and 343 on the RBD followed by refinement with ISOLDE (*42*). These coordinates were then prepared using QuickPrep (MOE v2020.0901, https://www.chemcomp.com). The Omicron RBD:hACE2:S304:S309 structure from PDB 7TN0 and Beta RBD:hACE2:S304:S309 structure determined here were glycosylated as described above and prepared using QuickPrep. The Wuhan-Hu-1+Q498R RBD:hACE2 and Omicron+Y501N RBD:hACE2:S304:S309 structures were generated by mutating the sidechain of Q498 to R and Y501 to N respectively followed by selecting the lowest energy rotamer (in MOE) for the mutated residues; further refinement was performed by excluding these residues from the restraint masks during minimization and equilibration (see below)

The coordinates of each complex were parameterized using tleap (*46*). The following force fields were used: Amber ff14SB for the protein (*47*), GLYCAM_06j-1 for glycans (*48*), TIP3P for water (*49*) and for the neutralizing 0.15 M of NaCl, Joung & Cheatham parameters (*50*) were used; the Li parameters (*51*) for Zn and divalent ions.

For each complex, a nine-stage restrained minimization and equilibration protocol was used as previously described (*52*) using Amber20 (*46*). Initial minimization involved 10,000 steps and positional restraints applied to all heavy atoms resolved in the crystal structures with a 100 kcal/molÅ^2 force constant. 8 independent simulations were initialized using different initial velocities drawn randomly from the Maxwell-Boltzmann distribution, in an NVT ensemble, gentle heating from 100 K to 300 K over 100 ps, and positional restraints on all heavy atoms with a 100 kcal/molÅ^2 force constant. Next the ensemble was switched to NPT, a constant temperature of 300 K over 100 ps, and positional restraints on all heavy atoms with a 100 kcal/molÅ^2 force constant. Next, a 250 ps stage was run in NPT at 300 K with positional restraints on all heavy atoms with a 10 kcal/molÅ^2 force constant. Next minimization for 10,000 steps was performed with positional restraints on backbone atoms with a 10 kcal/molÅ^2 force constant. This was followed by 100 ps of NPT at 300 K with positional restraints on backbone atoms with a 10 kcal/molÅ^2 force constant. Next 100 ps of NPT at 300 K with positional restraints on backbone atoms with a 1.0 kcal/molÅ^2 force constant. Next 100 ps of NPT at 300 K with positional restraints on backbone atoms with a 0.1 kcal/molÅ^2 force constant. The final stage of equilibration was 2.5 ns of unrestrained MD in NPT at 300 K for each of the 8 independent trajectories for each complex. The 8 pairs of simulations were extended for 700 ns each in NPT at 300 K. Snapshots were saved every 1 ns for post-processing. 5.6 μs of aggregated MD simulations were obtained for Omicron RBD:hACE2:S304:309 and 5.6 μs for Wuhan-Hu-1 RBD:hACE2.

Simulations were post-processed using cpptraj (*53*). Coordinates were imaged, RMS-aligned using Cα atoms to the respective crystal structure coordinates, water and ions stripped and saved to netcdf files. Distances were computed using the atoms of Y (HD1/2, HE1/2, HH), Q (OE1, HE1/2, NE2), N (OD1, HD1/2), D (OD1 or OD2), E (OE1 or OE2), R (HE, HH11/2, HH21/2), and K (HZ1/2/3). For each residue pair (H-bond or salt bridge) all distance pairs between atoms were computed and the lowest value of those pairs used as the hydrogen bond or salt bridge distance. Figures were generated using matplotlib.

Volumetric maps were computed using the VolMap Plugin in VMD (*54*) [Humphrey, W. F., Dalke, A. & Schulten, K. (1996) *J. Mol. Graphics* **14,** 33–38.]. Default parameters were used to compute the atomic densities observed over a grid, where the width of gaussian functions centered at each grid point bore widths equal to the atomic radii in each respective residue and then weighted by the atomic mass. The double sum of these gaussians over the grid points and over the course of the simulation were used to generate an isosurface map. The isosurfaces were rendered using an occupancy threshold of 0.5.

Comparisons among variant RBD structures

We analyzed atomic displacements between structures of ACE2-bound variant RBDs. X-ray crystallography structures included Wuhan-Hu-1: PDB 6M0J (*25*); Alpha: PDB 7EKF (*26*); Beta: PDB 7EKG (*26*); and Delta: PDB 7WBQ (*55*). Cryo-EM structure local refinements included Wuhan-Hu-1: PDB 7KMB (*56*); Alpha: PDB (*57*); Beta: PDB 7VX4 (*58*); and Delta: PDB 7V8B. Structures were aligned in PyMol to minimize RBD structure RMSD. Pairwise

distances between Cα or all-atom-averages (for unmutated sites) between aligned structures were computed using the bio3d package in R (*59*).

Data visualization

Interactive visualizations available at https://jbloomlab.github.io/SARS-CoV-2-RBD_DMS_variants/ were built using altair, version 4.2 (*60*). Sites of strong antibody escape (Fig. 2A and Fig. S4A) were those with an average normalized total escape of >0.05 across all antibodies in the aggregate tool described by (*11*) as of November 27, 2021 (https://raw.githubusercontent.com/jbloomlab/SARS2_RBD_Ab_escape_maps/03910f5bb6bc86ab823e9cf40f34b07b403f26d2/processed_data/escape_data.csv).

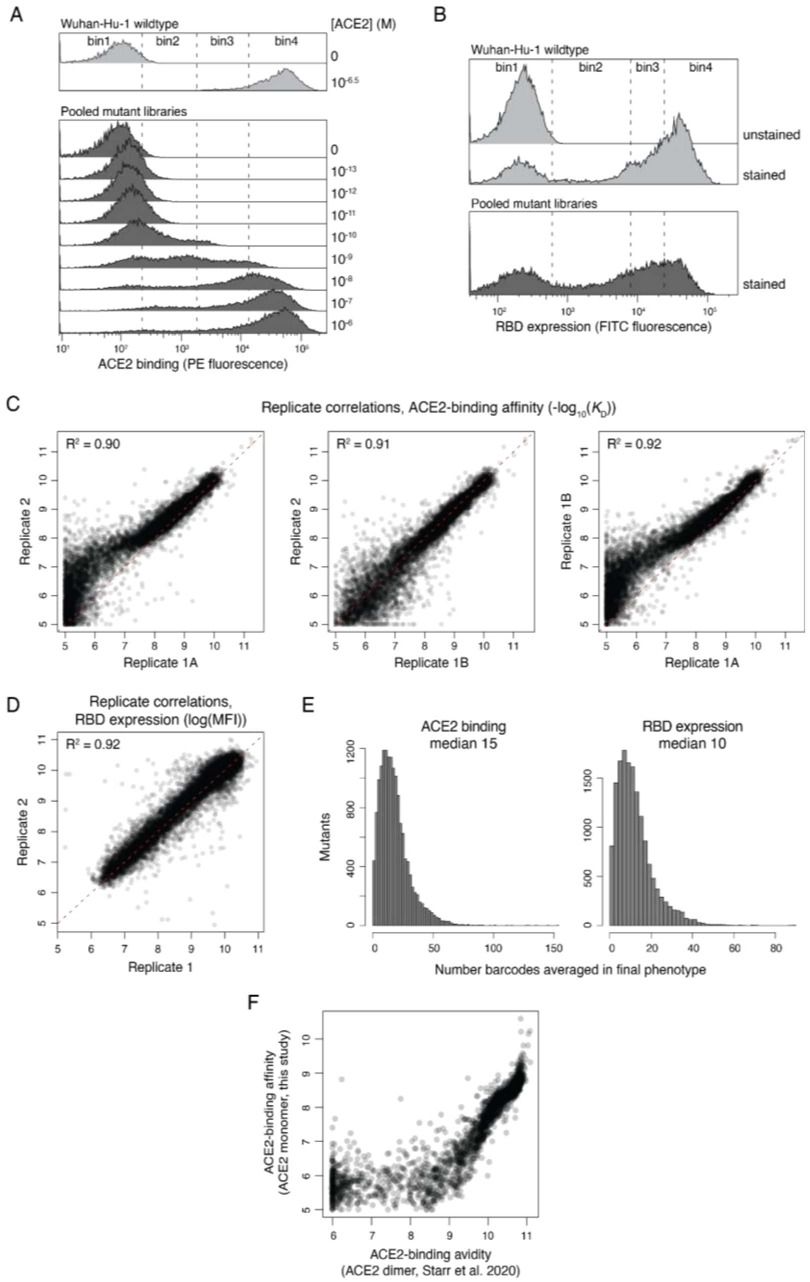

Fig. S1. Deep mutational scanning experimental details. (**A**) Representative flow cytometry traces and gates used in ACE2 binding titration FACS-seq assays on the pooled variant libraries. Gates were drawn to isolate single, RBD-positive cells, followed by binning on histograms of ACE2 binding (PE fluorescence) as shown. **(B)** Representative flow cytometry traces and gates used in RBD expression FACS-seq assays on the pooled variant libraries. Gates were drawn to isolate single cells, followed by binning on histograms of RBD expression (FITC fluorescence) as shown. (**C**) Correlations in mutant affinities as measured in triplicate ACE2 titration assays with pooled variant libraries. Red line, 1:1. Replicates 1A and 1B are independent experimental replicates performed on a single library, and replicate 2 is an experiment on an independently generated replicate library. (**D**) Correlations in mutant RBD expression levels as measured in duplicate Sort-seq assays with pooled variant libraries. Red line, 1:1. (**E**). Histograms of the number of barcodes that were averaged for the calculation of final deep mutational scanning scores for each amino acid mutant. (**F**) Relationship between the Wuhan-Hu-1 mutant ACE2-binding affinities as measured in the current study with monomeric ACE2 versus the prior avidity values measured in Starr et al. 2020 using dimeric ACE2 (*2*). Monomeric ligand unmasks affinity-enhancing effects that can be masked by avidity with dimeric ligand, while dimeric ligand exhibits a larger dynamic range over moderate-to-low affinity variants.

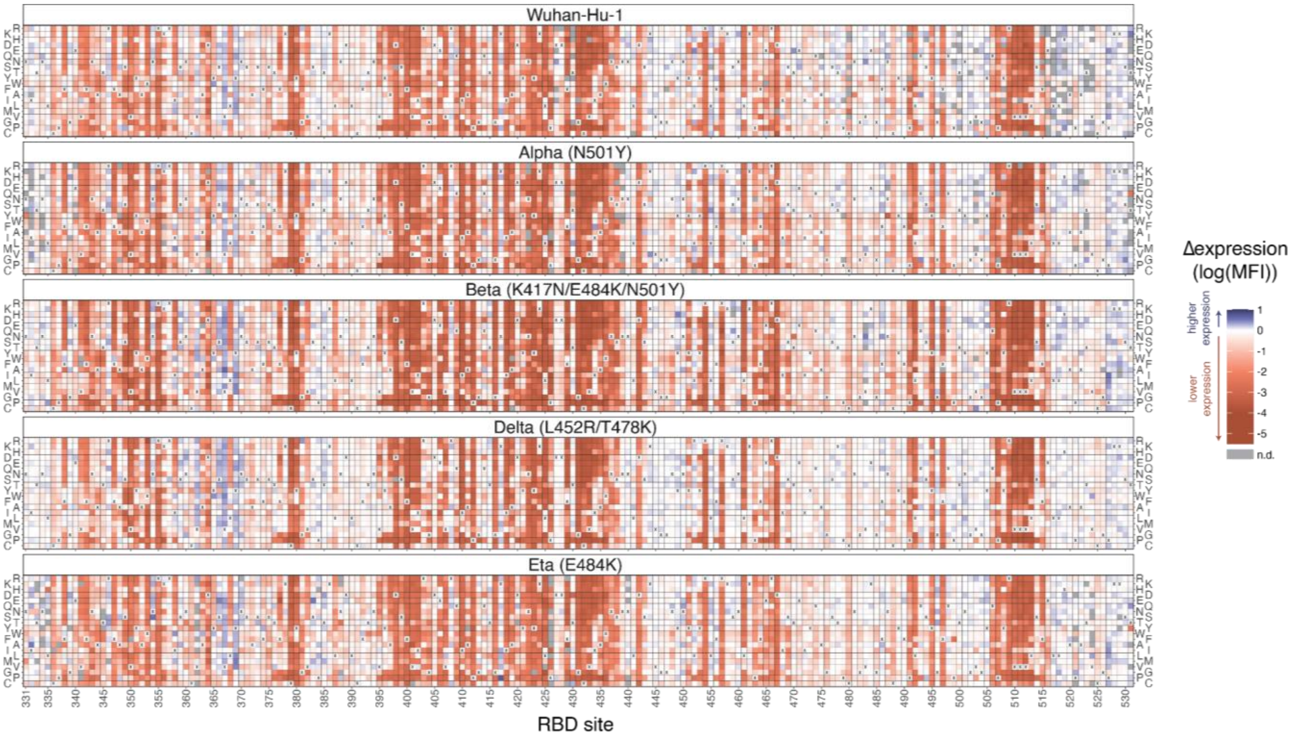

Fig. S2. Deep mutational scanning maps of mutational effects on RBD expression. The impact of every single amino-acid mutation in SARS-CoV-2 variant RBDs on yeast surface-expression levels as determined by FACS-seq assays (Fig. S1). The wildtype amino acid in each variant is indicated with an "x", and gray squares indicate missing mutations in each library. An interactive version of this map is at https://jbloomlab.github.io/SARS-CoV-2-RBD_DMS_variants/RBD-heatmaps/, and raw data are in Data S1.

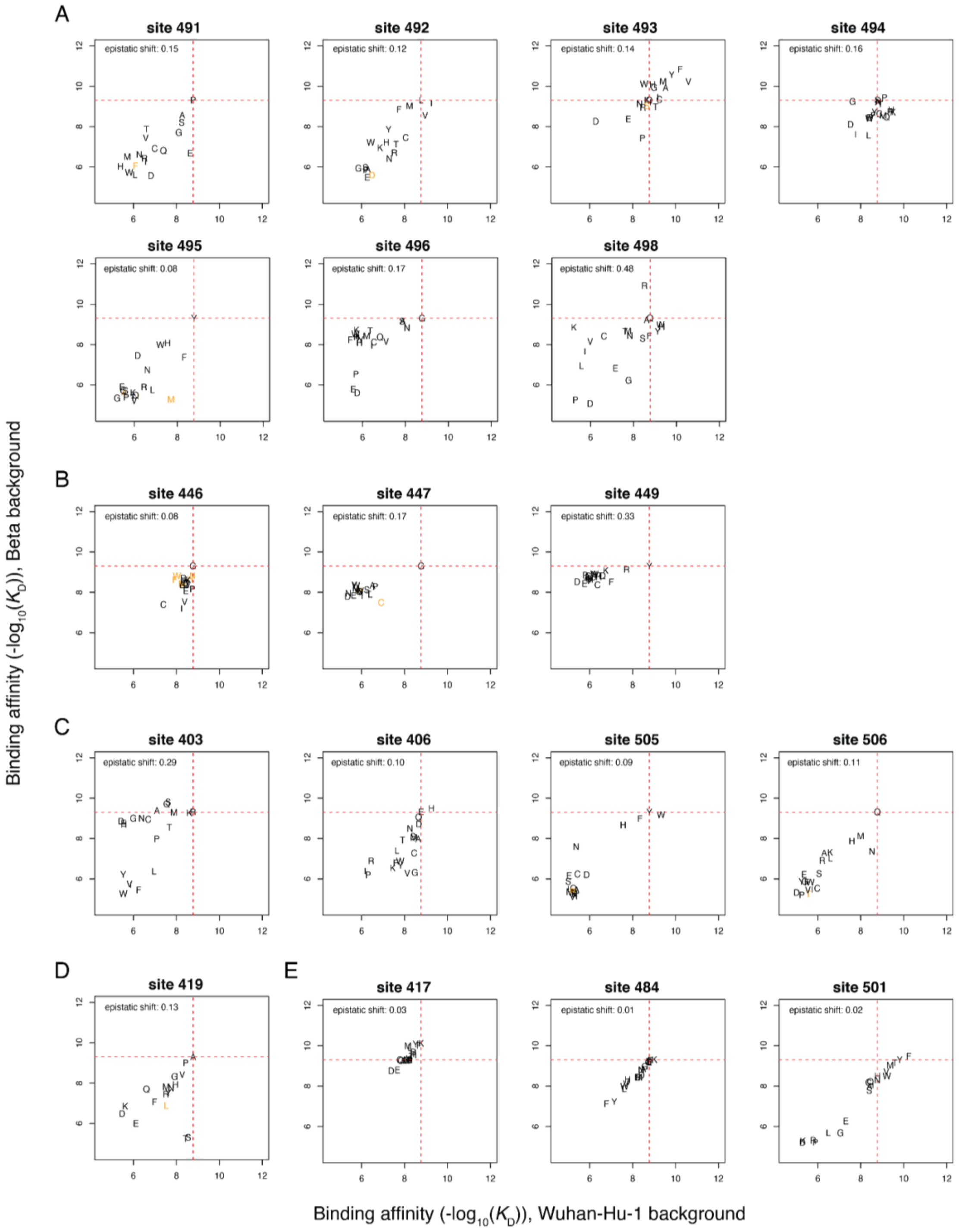

Fig. S3. Mutation-level perturbations at sites of interest between the Beta and Wuhan-Hu-1 RBDs. Scatter plots of mutation-level affinities for sites of interest: (**A**) 498 and the central ACE2-contact strand, (**B**) 446-449 loop, (**C**) site 403 and related sites, (**D**) site 419 where mutations to S/T add a glycan in the Beta RBD containing K417N, (**E**) and additive interaction among the sites mutated between Wuhan-Hu-1 and Beta. Details as in Fig. 2C. Orange letters are mutations that were sampled with fewer than 3 unique barcodes across titration replicates in the Beta and/or Wuhan-Hu-1 data and therefore not included in the epistatic shift computation as described in Methods. Scatterplots for all sites can be visualized at https://jbloomlab.github.io/SARS-CoV-2-RBD_DMS_variants/epistatic-shifts/.

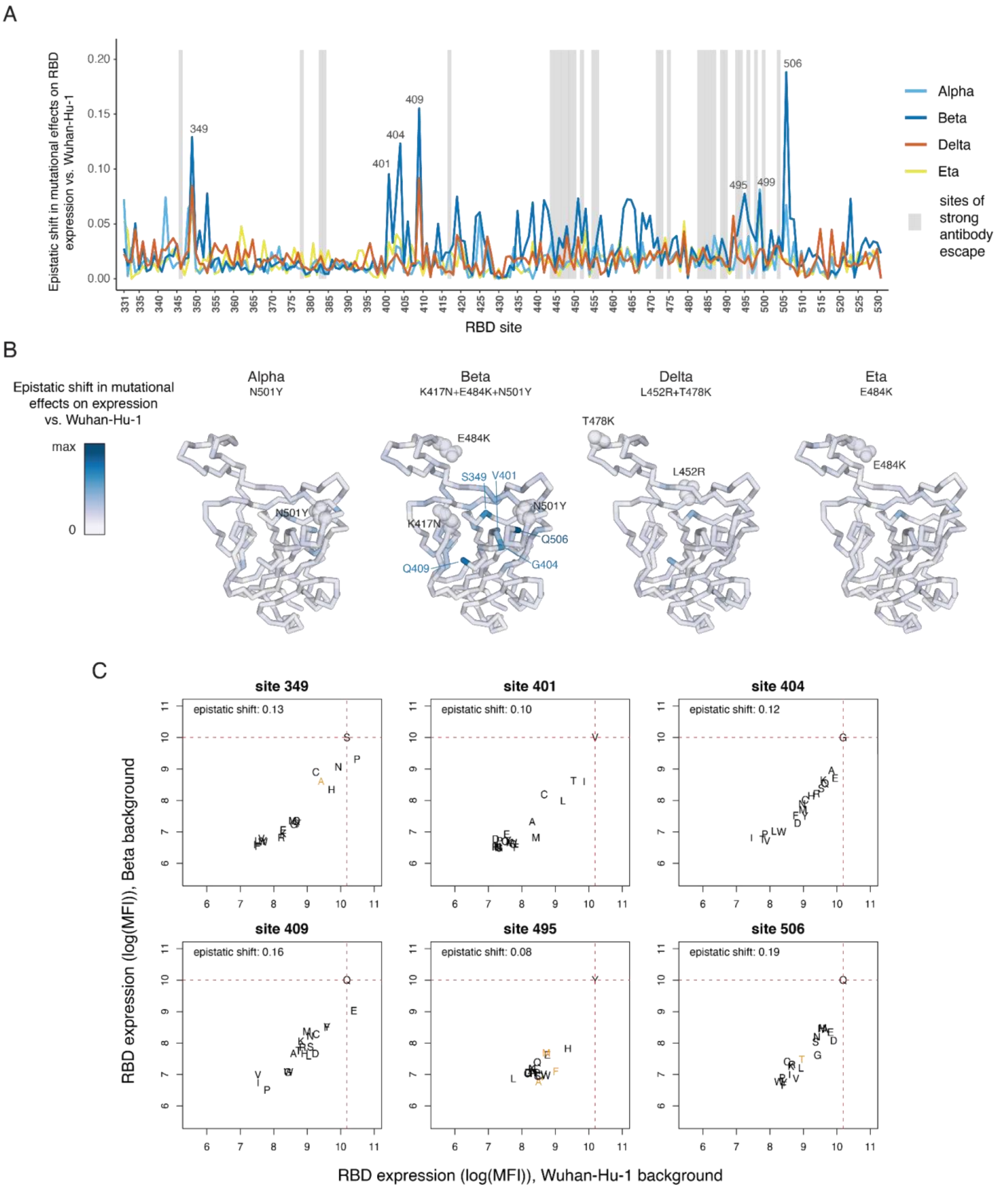

Fig. S4. Epistatic shifts in mutational impacts on RBD expression. (**A**) The epistatic shift in mutational effects on RBD expression at each RBD site between the indicated variant and Wuhan-Hu-1. The epistatic shift is calculated as in Fig. 2A (see Methods). (**B**) Structural projection of the epistatic shifts onto the backbone of the Wuhan-Hu-1 RBD structure (PDB 6M0J). Details as in Fig. 2B. (**C**) Scatters of mutation-level RBD expression values for sites of strong epistatic shifts in (B). Details as in Fig. 2C and Fig. S3. These scatter plots suggest that at the shifted sites, the Beta RBD is more sensitive to mutation compared to Wuhan-Hu-1, reflected in the concave shape of each plot. This is in contrast to idiosyncratic epistatic shifts in individual mutation's effects on ACE2 binding as seen in e.g. Fig. 2C.

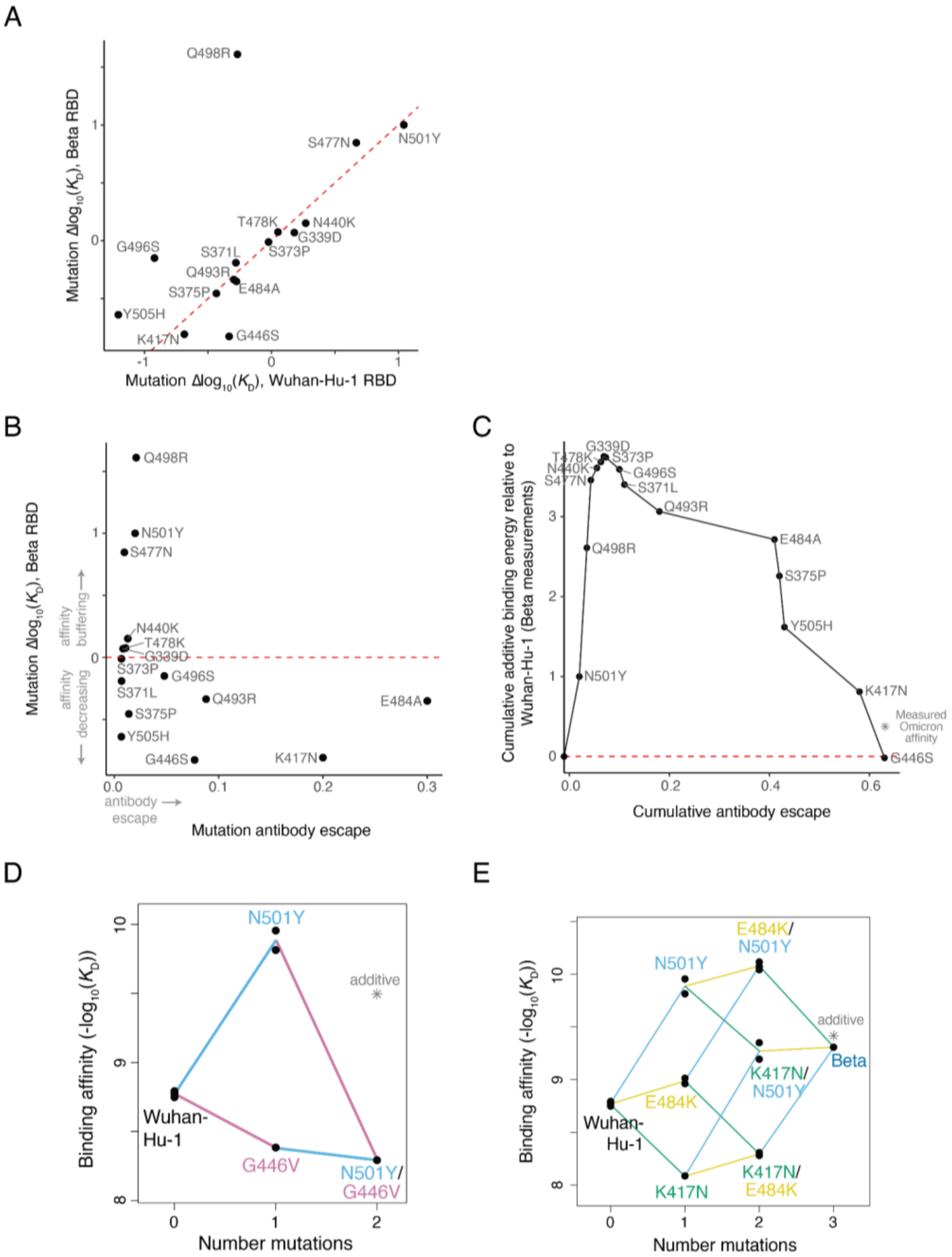

Fig. S5. Epistasis and affinity-buffering of Omicron BA.1 RBD mutations. (**A**) Scatter plot illustrating the difference in effect on ACE2 binding for each of the 15 Omicron BA.1 mutations (Fig. 3B) when introduced in the Beta versus Wuhan-Hu-1 RBD. Red line, 1:1. (**B**) Relationship between mutation effects on ACE2 binding versus antibody escape for the fifteen Omicron BA.1 RBD mutations (Fig. 3B). Antibody escape is estimated from a calculator that aggregates >250 antibody deep mutational scanning escape profiles to determine the expected antigenic effect of mutations (*11*). (**C**) Cumulative ACE2-binding affinity versus antibody escape of Omicron BA.1 mutations (Fig. 3B and (B)). Antibody escape calculated as in (B), except mutations were introduced consecutively into the calculator in the order shown, which accounts for possible redundancy in escape mutations at overlapping antibody epitopes. (**D**) Double mutant cycle diagram illustrating negative sign epistasis between N501Y and G446V. (**E**) Triple mutant cycle illustrating additivity among the three mutations in the Beta RBD. See also Fig. S4E.

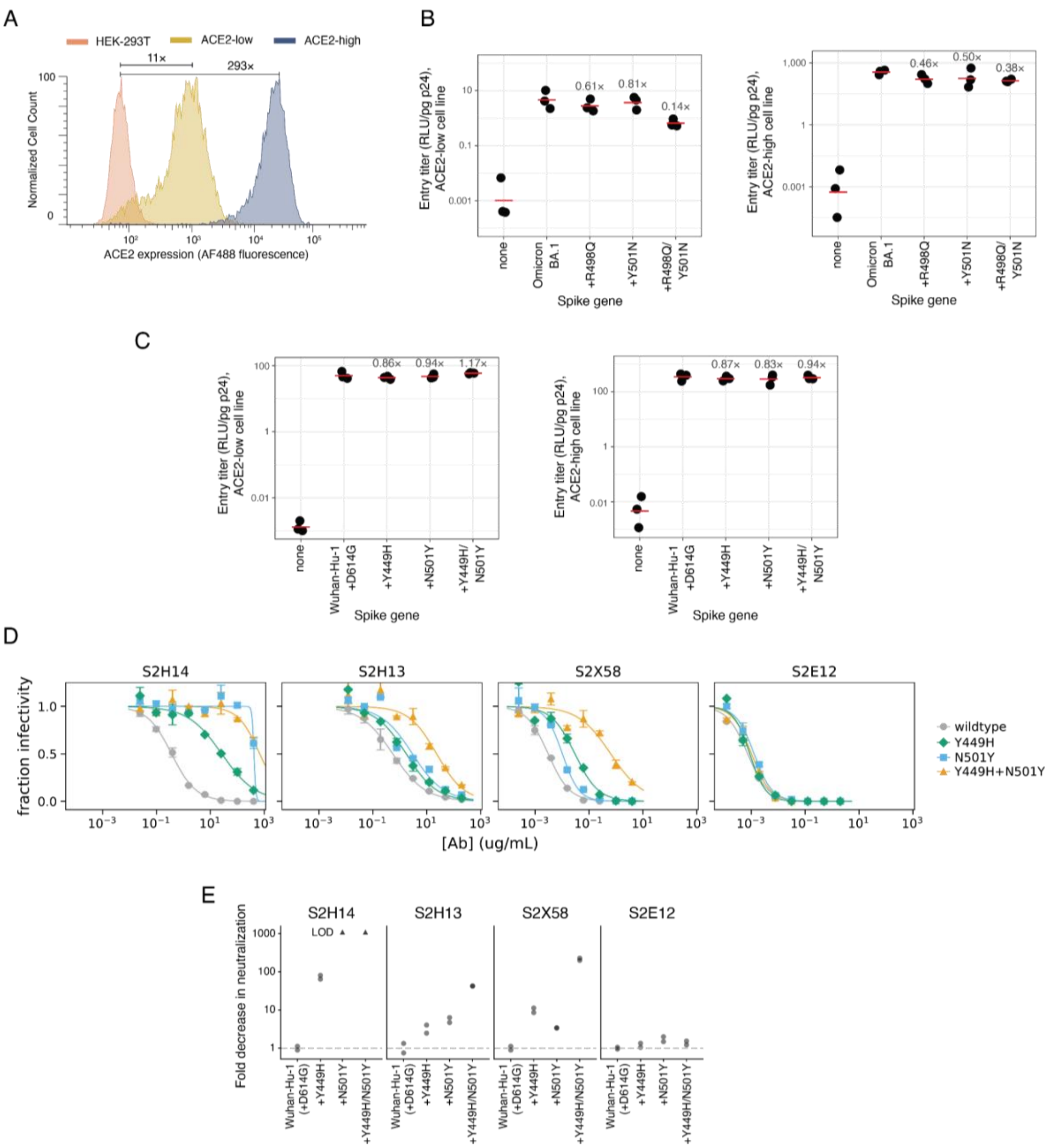

Fig. S6. Pseudoviral entry and neutralization assays. (**A**) ACE2 expression of HEK-293T cell lines, determined by antibody labeling of surface ACE2 expression and flow cytometry. Label indicates fold-increase in fluorescence geometric mean. (**B**) Normalized entry titers of Omicron BA.1 (or reversion mutant) spike-pseudotyped lentiviral particles on HEK-293T cell lines expressing low (left; independent titering replicate of Fig. 3C) or high (right) levels of ACE2. Labels indicate fold-change in geometric mean entry (red bar) across biological triplicate measurements. (**C**) Normalized entry titers of Wuhan-Hu-1+D614G (and RBD mutant) spike-pseudotyped lentiviral particles on HEK-293T cell lines expressing low (left) or high (right) levels of ACE2. (**D**) Neutralization of spike-pseudotyped lentiviral particles by human monoclonal antibodies S2H14, S2H13, and S2X58 that showed escape at site Y449 in prior deep mutational scanning data (*18*). Each point is the mean and standard error from technical duplicates. S2E12 is a negative control antibody, which was not expected to be escaped by the Y449H or N501Y mutations based on prior deep mutational scanning data (*18*). (**E**) Fold-change in neutralization of spike-pseudotyped lentivirus from the curves in (D). The Y449H/N501Y double mutant shows 2.4x and 6.3x synergistic escape from S2H13 and S2X58, respectively, compared to the multiplicative fold-change of the single mutants.

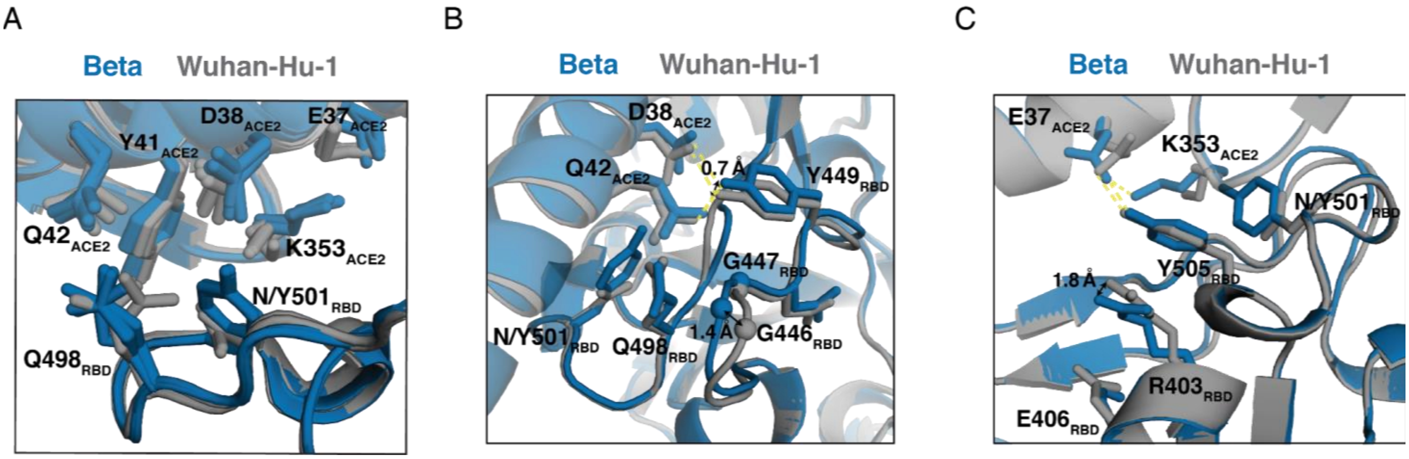

Fig. S7. Structural comparison of epistatically shifted sites between Wuhan-Hu-1 and Beta RBDs. Zoomed structural views of clusters of epistatically shifted sites visualized in Wuhan-Hu-1 and Beta ACE2-bound RBD X-ray crystal structures. (**A**) Site 498 shows dramatic epistatic shifts in the presence of N501Y. Comparison of multiple crystal structures of Wuhan-Hu-1 (PDBs 6M0J, 6VW1 [Wuhan-Hu-1 RBM chimera on SARS-CoV-1 RBD scaffold]) and Beta (this study and PDB 7EKG) illustrates overlapping heterogeneity in Q498 rotamers between Wuhan-Hu-1 and Beta structures. (**B**) Sites 446, 447, and 449 exhibit small shifts in backbone and side chain positions in the ACE2-bound structure, though Delta exhibits similar variability in backbone and sidechain positions (see Fig S8B,C) despite lacking epistatic shifts at these positions. (**C**) The epistatically shifted residues 505, 403, and 406 show little structural perturbation despite large shifts in mutational effects, especially at residue 403 (see Fig. 2C). Site 403 has not previously been implicated in key events in the functional or antigenic evolution of SARS-related coronaviruses, but mutations in this region have been shown to transmit allosterically to distal regions of the ACE2-binding surface (*61*, *62*), which may be further reflected in many epistatic shifts in mutation effects on RBD expression in this region (Fig. S4).

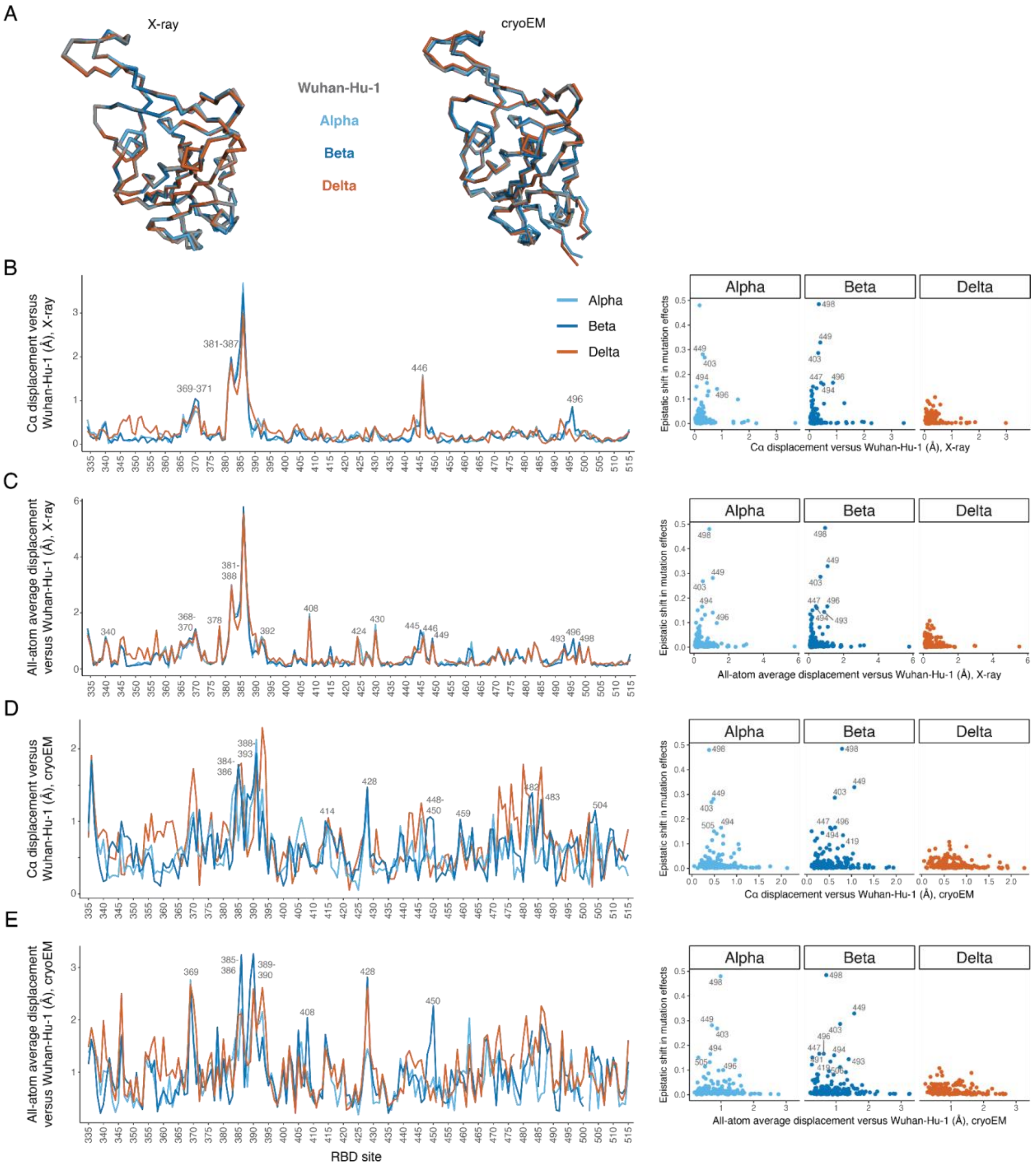

Fig. S8. Structural perturbations among variant RBD structures. (**A**) Backbone structure alignment between ACE2-bound RBD X-ray crystal structures (left; Wuhan-Hu-1 (PDB 6M0J, 2.45Å resolution), Alpha (7EKF, 2.85Å), Beta (7EKG, 2.63Å), and Delta (7WBQ, 3.34Å)) or local refinement maps from ACE2-bound spike cryo-EM structures (right; Wuhan-Hu-1 (PDB 7KMB, 3.39Å resolution), Alpha (7MJN, 3.29Å), Beta (7VX4, 3.90Å) and Delta (7V8B, 3.2Å)). (**B**-**E**) Changes in backbone and sidechain properties in variant versus Wuhan-Hu-1 structures. For each property, the left hand plot shows the value for each variant RBD versus Wuhan-Hu-1 as a lineplot across RBD sites; sites with the largest displacements in the Beta structures are labeled. Right Hand plot shows relationship across RBD sites between the property and epistatic shift from deep mutational scanning (Fig. 2A). Calculated properties include the mainchain Cα displacement from X-ray (B) or cryoEM (D) structures, and all-atom average displacement from X-ray (C) or cryoEM (E) structures.

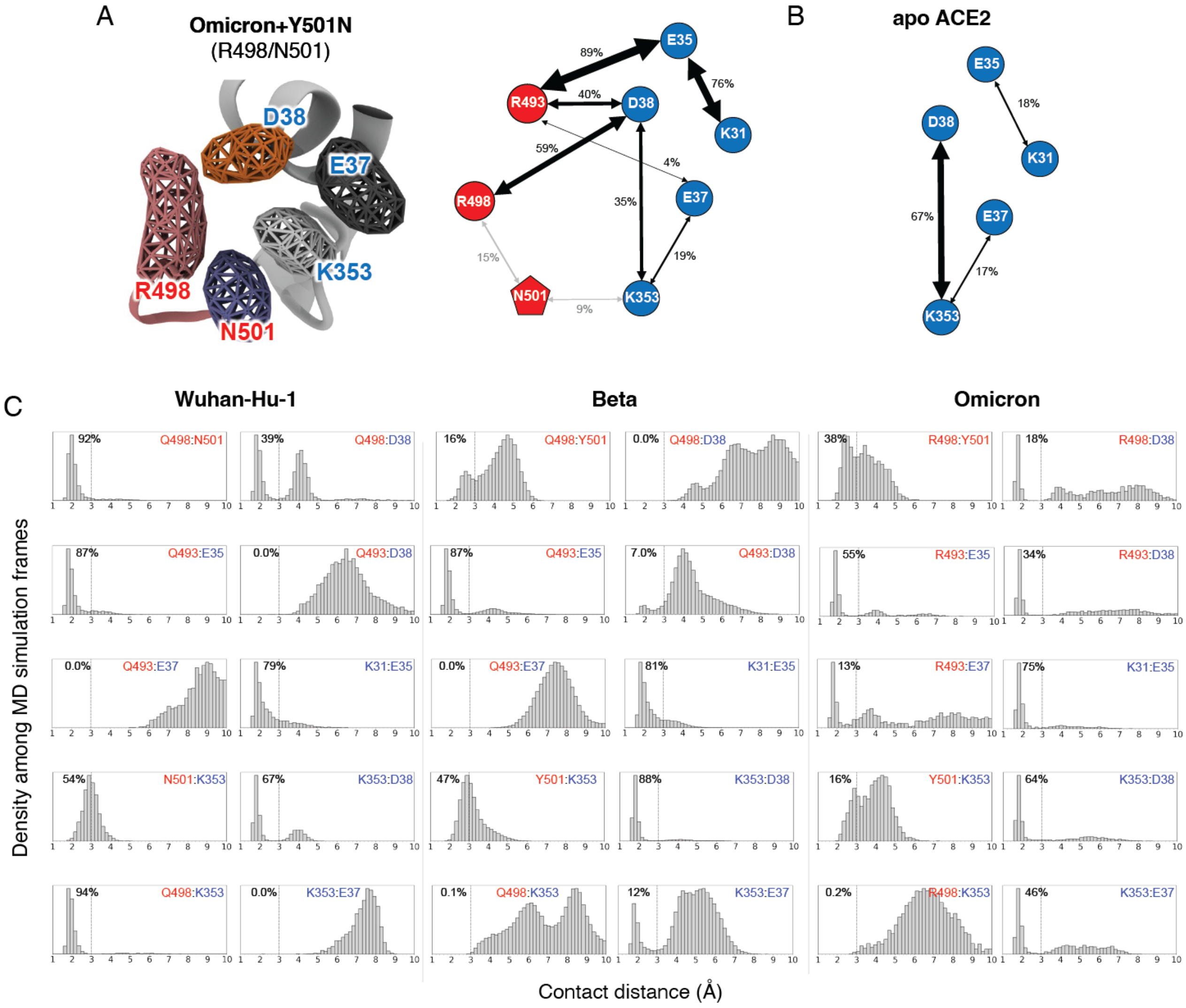

Fig. S9. Dynamic RBD:ACE2 interface as revealed in molecular dynamics simulation. (**A**, **B**) Equivalent diagrams to Fig. 4C for Omicron+Y501N (A) and apo ACE2 (B). (**C**) Histograms showing the distances between polar contact donors/acceptors over the course of molecular dynamics simulation of Wuhan-Hu-1 (left), Beta (middle), or Omicron (right) RBD bound to ACE2, with the percentage of frames where residues are within 3.0Å distance (dashed line) labeled.

Table S1. Crystallographic data collection and refinement statistics.

	Beta RBD/ACE2/S304/S309<br>PDB
**Data collection**	
Space group	P1
Cell dimensions	
a, *b*, *c* (Å)	78.53, 126.77, 145.66
α, β, γ (°)	91.78, 103.66, 96.02
Resolution (Å)	63.19-2.45 (2.49-2.45)
R_{merge}	0.119 (1.862)
R_{pim}	0.071 (1.092)
$I / \sigma I$	6.2 (0.5)
Completeness (%)	92.2 (93.4)
Redundancy	3.7 (3.8)
**Refinement**	
Resolution (Å)	2.45
No. reflections	180,906
R_{work} / R_{free}	0.199/0.233
No. atoms	
Protein	26,009
Ligand/ion	811
Water	1,085
B-factors	
Protein	72.84
Ligand/ion	85.48
Water	62.21
R.m.s. deviations	
Bond lengths (Å)	0.009
Bond angles (°)	1.14
Ramachandran	
Favored (%)	97.5
Outliers (%)	0.1

*Values in parentheses are for highest-resolution shell.

Data S1. (separate file)

Raw data containing the effect of each mutation in each RBD on ACE2-binding affinity and RBD expression. `bind` (and `delta_bind`) represent the -$\log_{10}(K_D)$ of a mutant (and $\Delta\log_{10}(K_D)$ relative to the respective parental sequence). `expr` (and `delta_expr`) represent the log(MFI) of a mutation (and Δlog(MFI) relative to the respective parental sequence). Additional table elements give the number of unique experimental replicates and number of internally replicated barcode sequences with which each mutant phenotype was determined.

References and Notes

1. K. Tao, P. L. Tzou, J. Nouhin, R. K. Gupta, T. de Oliveira, S. L. Kosakovsky Pond, D. Fera, R. W. Shafer, The biological and clinical significance of emerging SARS-CoV-2 variants. *Nat. Rev. Genet.* **22**, 757–773 (2021). doi:10.1038/s41576-021-00408-x Medline

2. T. N. Starr, A. J. Greaney, S. K. Hilton, D. Ellis, K. H. D. Crawford, A. S. Dingens, M. J. Navarro, J. E. Bowen, M. A. Tortorici, A. C. Walls, N. P. King, D. Veesler, J. D. Bloom, Deep Mutational Scanning of SARS-CoV-2 Receptor Binding Domain Reveals Constraints on Folding and ACE2 Binding. *Cell* **182**, 1295–1310.e20 (2020). doi:10.1016/j.cell.2020.08.012 Medline

3. Y. Liu, J. Liu, K. S. Plante, J. A. Plante, X. Xie, X. Zhang, Z. Ku, Z. An, D. Scharton, C. Schindewolf, S. G. Widen, V. D. Menachery, P.-Y. Shi, S. C. Weaver, The N501Y spike substitution enhances SARS-CoV-2 infection and transmission. *Nature* **602**, 294–299 (2022). doi:10.1038/s41586-021-04245-0 Medline

4. T. N. Starr, J. W. Thornton, Epistasis in protein evolution. *Protein Sci.* **25**, 1204–1218 (2016). doi:10.1002/pro.2897 Medline

5. T. N. Starr, S. K. Zepeda, A. C. Walls, A. J. Greaney, S. Alkhovsky, D. Veesler, J. D. Bloom, ACE2 binding is an ancestral and evolvable trait of sarbecoviruses. *Nature* **603**, 913–918 (2022). doi:10.1038/s41586-022-04464-z Medline

6. J. Zahradník, S. Marciano, M. Shemesh, E. Zoler, D. Harari, J. Chiaravalli, B. Meyer, Y. Rudich, C. Li, I. Marton, O. Dym, N. Elad, M. G. Lewis, H. Andersen, M. Gagne, R. A. Seder, D. C. Douek, G. Schreiber, SARS-CoV-2 variant prediction and antiviral drug design are enabled by RBD in vitro evolution. *Nat. Microbiol.* **6**, 1188–1198 (2021). doi:10.1038/s41564-021-00954-4 Medline

7. N. Bate, C. G. Savva, P. C. E. Moody, E. A. Brown, J. K. Ball, J. W. R. Schwabe, J. E. Sale, N. P. J. Brindle, In vitro evolution predicts emerging CoV-2 mutations with high affinity for ACE2 and cross-species binding. bioRxiv 473975 [Preprint] (2021); doi:10.1101/2021.12.23.473975.

8. R. Viana, S. Moyo, D. G. Amoako, H. Tegally, C. Scheepers, C. L. Althaus, U. J. Anyaneji, P. A. Bester, M. F. Boni, M. Chand, W. T. Choga, R. Colquhoun, M. Davids, K. Deforche, D. Doolabh, L. du Plessis, S. Engelbrecht, J. Everatt, J. Giandhari, M. Giovanetti, D. Hardie, V. Hill, N.-Y. Hsiao, A. Iranzadeh, A. Ismail, C. Joseph, R. Joseph, L. Koopile, S. L. Kosakovsky Pond, M. U. G. Kraemer, L. Kuate-Lere, O. Laguda-Akingba, O. Lesetedi-Mafoko, R. J. Lessells, S. Lockman, A. G. Lucaci, A. Maharaj, B. Mahlangu, T. Maponga, K. Mahlakwane, Z. Makatini, G. Marais, D. Maruapula, K. Masupu, M. Matshaba, S. Mayaphi, N. Mbhele, M. B. Mbulawa, A. Mendes, K. Mlisana, A. Mnguni, T. Mohale, M. Moir, K. Moruisi, M. Mosepele, G. Motsatsi, M. S. Motswaledi, T. Mphoyakgosi, N. Msomi, P. N. Mwangi, Y. Naidoo, N. Ntuli, M. Nyaga, L. Olubayo, S. Pillay, B. Radibe, Y. Ramphal, U. Ramphal, J. E. San, L. Scott, R. Shapiro, L. Singh, P. Smith-Lawrence, W. Stevens, A. Strydom, K. Subramoney, N. Tebeila, D. Tshiabuila, J. Tsui, S. van Wyk, S. Weaver, C. K. Wibmer, E. Wilkinson, N. Wolter, A. E. Zarebski, B. Zuze, D. Goedhals, W. Preiser, F. Treurnicht, M. Venter, C. Williamson, O. G. Pybus, J. Bhiman, A. Glass, D. P. Martin, A. Rambaut, S. Gaseitsiwe, A. von Gottberg, T. de

Oliveira, Rapid epidemic expansion of the SARS-CoV-2 Omicron variant in southern Africa. *Nature* **603**, 679–686 (2022). doi:10.1038/s41586-022-04411-y Medline

9. K. Javanmardi, C.-W. Chou, C. I. Terrace, A. Annapareddy, T. S. Kaoud, Q. Guo, J. Lutgens, H. Zorkic, A. P. Horton, E. C. Gardner, G. Nguyen, D. R. Boutz, J. Goike, W. N. Voss, H.-C. Kuo, K. N. Dalby, J. D. Gollihar, I. J. Finkelstein, Rapid characterization of spike variants via mammalian cell surface display. *Mol. Cell* **81**, 5099–5111.e8 (2021). doi:10.1016/j.molcel.2021.11.024 Medline

10. K. K. Chan, T. J. C. Tan, K. K. Narayanan, E. Procko, An engineered decoy receptor for SARS-CoV-2 broadly binds protein S sequence variants. *Sci. Adv.* **7**, eabf1738 (2021). doi:10.1126/sciadv.abf1738 Medline

11. A. J. Greaney, T. N. Starr, J. D. Bloom, An antibody-escape calculator for mutations to the SARS-CoV-2 receptor-binding domain. bioRxiv 471236 [Preprint] (2021), doi:10.1101/2021.12.04.471236.

12. E. Cameroni, J. E. Bowen, L. E. Rosen, C. Saliba, S. K. Zepeda, K. Culap, D. Pinto, L. A. VanBlargan, A. De Marco, J. di Iulio, F. Zatta, H. Kaiser, J. Noack, N. Farhat, N. Czudnochowski, C. Havenar-Daughton, K. R. Sprouse, J. R. Dillen, A. E. Powell, A. Chen, C. Maher, L. Yin, D. Sun, L. Soriaga, J. Bassi, C. Silacci-Fregni, C. Gustafsson, N. M. Franko, J. Logue, N. T. Iqbal, I. Mazzitelli, J. Geffner, R. Grifantini, H. Chu, A. Gori, A. Riva, O. Giannini, A. Ceschi, P. Ferrari, P. E. Cippà, A. Franzetti-Pellanda, C. Garzoni, P. J. Halfmann, Y. Kawaoka, C. Hebner, L. A. Purcell, L. Piccoli, M. S. Pizzuto, A. C. Walls, M. S. Diamond, A. Telenti, H. W. Virgin, A. Lanzavecchia, G. Snell, D. Veesler, D. Corti, Broadly neutralizing antibodies overcome SARS-CoV-2 Omicron antigenic shift. *Nature* **602**, 664–670 (2022). doi:10.1038/s41586-021-04386-2 Medline

13. B. Meng, A. Abdullahi, I. A. T. M. Ferreira, N. Goonawardane, A. Saito, I. Kimura, D. Yamasoba, P. P. Gerber, S. Fatihi, S. Rathore, S. K. Zepeda, G. Papa, S. A. Kemp, T. Ikeda, M. Toyoda, T. S. Tan, J. Kuramochi, S. Mitsunaga, T. Ueno, K. Shirakawa, A. Takaori-Kondo, T. Brevini, D. L. Mallery, O. J. Charles, J. E. Bowen, A. Joshi, A. C. Walls, L. Jackson, D. Martin, K. G. C. Smith, J. Bradley, J. A. G. Briggs, J. Choi, E. Madissoon, K. B. Meyer, P. Mlcochova, L. Ceron-Gutierrez, R. Doffinger, S. A. Teichmann, A. J. Fisher, M. S. Pizzuto, A. de Marco, D. Corti, M. Hosmillo, J. H. Lee, L. C. James, L. Thukral, D. Veesler, A. Sigal, F. Sampaziotis, I. G. Goodfellow, N. J. Matheson, K. Sato, R. K. Gupta, CITIID-NIHR BioResource COVID-19 Collaboration, Genotype to Phenotype Japan (G2P-Japan) Consortium, Ecuador-COVID19 Consortium, Altered TMPRSS2 usage by SARS-CoV-2 Omicron impacts infectivity and fusogenicity. *Nature* **603**, 706–714 (2022). doi:10.1038/s41586-022-04474-x Medline

14. M. McCallum, N. Czudnochowski, L. E. Rosen, S. K. Zepeda, J. E. Bowen, A. C. Walls, K. Hauser, A. Joshi, C. Stewart, J. R. Dillen, A. E. Powell, T. I. Croll, J. Nix, H. W. Virgin, D. Corti, G. Snell, D. Veesler, Structural basis of SARS-CoV-2 Omicron immune evasion and receptor engagement. *Science* **375**, 864–868 (2022). doi:10.1126/science.abn8652 Medline

15. A. J. Greaney, T. N. Starr, C. O. Barnes, Y. Weisblum, F. Schmidt, M. Caskey, C. Gaebler, A. Cho, M. Agudelo, S. Finkin, Z. Wang, D. Poston, F. Muecksch, T. Hatziioannou, P.

D. Bieniasz, D. F. Robbiani, M. C. Nussenzweig, P. J. Bjorkman, J. D. Bloom, Mapping mutations to the SARS-CoV-2 RBD that escape binding by different classes of antibodies. *Nat. Commun.* **12**, 4196 (2021). doi:10.1038/s41467-021-24435-8 Medline

16. A. J. Greaney, T. N. Starr, R. T. Eguia, A. N. Loes, K. Khan, F. Karim, S. Cele, J. E. Bowen, J. K. Logue, D. Corti, D. Veesler, H. Y. Chu, A. Sigal, J. D. Bloom, A SARS-CoV-2 variant elicits an antibody response with a shifted immunodominance hierarchy. *PLOS Pathog.* **18**, e1010248 (2022). doi:10.1371/journal.ppat.1010248 Medline

17. F. Schmidt, Y. Weisblum, M. Rutkowska, D. Poston, J. DaSilva, F. Zhang, E. Bednarski, A. Cho, D. J. Schaefer-Babajew, C. Gaebler, M. Caskey, M. C. Nussenzweig, T. Hatziioannou, P. D. Bieniasz, High genetic barrier to SARS-CoV-2 polyclonal neutralizing antibody escape. *Nature* **600**, 512–516 (2021). doi:10.1038/s41586-021-04005-0 Medline

18. T. N. Starr, N. Czudnochowski, Z. Liu, F. Zatta, Y.-J. Park, A. Addetia, D. Pinto, M. Beltramello, P. Hernandez, A. J. Greaney, R. Marzi, W. G. Glass, I. Zhang, A. S. Dingens, J. E. Bowen, M. A. Tortorici, A. C. Walls, J. A. Wojcechowskyj, A. De Marco, L. E. Rosen, J. Zhou, M. Montiel-Ruiz, H. Kaiser, J. R. Dillen, H. Tucker, J. Bassi, C. Silacci-Fregni, M. P. Housley, J. di Iulio, G. Lombardo, M. Agostini, N. Sprugasci, K. Culap, S. Jaconi, M. Meury, E. Dellota Jr., R. Abdelnabi, S. C. Foo, E. Cameroni, S. Stumpf, T. I. Croll, J. C. Nix, C. Havenar-Daughton, L. Piccoli, F. Benigni, J. Neyts, A. Telenti, F. A. Lempp, M. S. Pizzuto, J. D. Chodera, C. M. Hebner, H. W. Virgin, S. P. J. Whelan, D. Veesler, D. Corti, J. D. Bloom, G. Snell, SARS-CoV-2 RBD antibodies that maximize breadth and resistance to escape. *Nature* **597**, 97–102 (2021). doi:10.1038/s41586-021-03807-6 Medline

19. C. Scheepers, J. Everatt, D. G. Amoako, H. Tegally, C. K. Wibmer, A. Mnguni, A. Ismail, B. Mahlangu, B. E. Lambson, S. I. Richardson, D. P. Martin, E. Wilkinson, J. E. San, J. Giandhari, N. Manamela, N. Ntuli, P. Kgagudi, S. Cele, S. Pillay, T. Mohale, U. Ramphal, Y. Naidoo, Z. T. Khumalo, G. Kwatra, G. Gray, L.-G. Bekker, S. A. Madhi, V. Baillie, W. C. Van Voorhis, F. K. Ngs-Sa, M. Treurnicht, M. Venter, K. Mlisana, N. Wolter, A. Sigal, C. Williamson, N.-Y. Hsiao, N. Msomi, T. Maponga, W. Preiser, Z. Makatini, R. Lessells, P. L. Moore, T. de Oliveira, A. von Gottberg, J. N. Bhiman, Emergence and phenotypic characterization of the global SARS-CoV-2 C.1.2 lineage. *Nat. Commun.* **13**, 1976 (2021). doi:10.1038/s41467-022-29579-9 Medline

20. T. Tada, H. Zhou, B. M. Dcosta, M. I. Samanovic, A. Cornelius, R. S. Herati, M. J. Mulligan, N. R. Landau, High-titer neutralization of Mu and C.1.2 SARS-CoV-2 variants by vaccine-elicited antibodies of previously infected individuals. *Cell Rep.* **38**, 110237 (2022). doi:10.1016/j.celrep.2021.110237 Medline

21. P. Colson, J. Delerce, E. Burel, J. Dahan, A. Jouffret, F. Fenollar, N. Yahi, J. Fantini, B. La Scola, D. Raoult, Emergence in Southern France of a new SARS-CoV-2 variant of probably Cameroonian origin harbouring both substitutions N501Y and E484K in the spike protein. bioRxiv 21268174 [Preprint] (2021); doi:10.1101/2021.12.24.21268174.

22. J. McBroome, B. Thornlow, A. S. Hinrichs, A. Kramer, N. De Maio, N. Goldman, D. Haussler, R. Corbett-Detig, Y. Turakhia, A daily-updated database and tools for

comprehensive SARS-CoV-2 mutation-annotated trees. *Mol. Biol. Evol.* **38**, 5819–5824 (2021). doi:10.1093/molbev/msab264 Medline

23. C. Laffeber, K. de Koning, R. Kanaar, J. H. G. Lebbink, Experimental Evidence for Enhanced Receptor Binding by Rapidly Spreading SARS-CoV-2 Variants. *J. Mol. Biol.* **433**, 167058 (2021). doi:10.1016/j.jmb.2021.167058 Medline

24. M. Yuan, D. Huang, C. D. Lee, N. C. Wu, A. M. Jackson, X. Zhu, H. Liu, L. Peng, M. J. van Gils, R. W. Sanders, D. R. Burton, S. M. Reincke, H. Prüss, J. Kreye, D. Nemazee, A. B. Ward, I. A. Wilson, Structural and functional ramifications of antigenic drift in recent SARS-CoV-2 variants. *Science* **373**, 818–823 (2021). doi:10.1126/science.abh1139 Medline

25. J. Lan, J. Ge, J. Yu, S. Shan, H. Zhou, S. Fan, Q. Zhang, X. Shi, Q. Wang, L. Zhang, X. Wang, Structure of the SARS-CoV-2 spike receptor-binding domain bound to the ACE2 receptor. *Nature* **581**, 215–220 (2020). doi:10.1038/s41586-020-2180-5 Medline

26. P. Han, C. Su, Y. Zhang, C. Bai, A. Zheng, C. Qiao, Q. Wang, S. Niu, Q. Chen, Y. Zhang, W. Li, H. Liao, J. Li, Z. Zhang, H. Cho, M. Yang, X. Rong, Y. Hu, N. Huang, J. Yan, Q. Wang, X. Zhao, G. F. Gao, J. Qi, Molecular insights into receptor binding of recent emerging SARS-CoV-2 variants. *Nat. Commun.* **12**, 6103 (2021). doi:10.1038/s41467-021-26401-w Medline

27. R. Eguia, K. H. D. Crawford, T. Stevens-Ayers, L. Kelnhofer-Millevolte, A. L. Greninger, J. A. Englund, M. J. Boeckh, J. D. Bloom, A human coronavirus evolves antigenically to escape antibody immunity. *PLOS Pathog.* **17**, e1009453 (2020). doi:10.1371/journal.ppat.1009453 Medline

28. A. H. M. Wong, A. C. A. Tomlinson, D. Zhou, M. Satkunarajah, K. Chen, C. Sharon, M. Desforges, P. J. Talbot, J. M. Rini, Receptor-binding loops in alphacoronavirus adaptation and evolution. *Nat. Commun.* **8**, 1735 (2017). doi:10.1038/s41467-017-01706-x Medline

29. Z. Li, A. C. Tomlinson, A. H. Wong, D. Zhou, M. Desforges, P. J. Talbot, S. Benlekbir, J. L. Rubinstein, J. M. Rini, The human coronavirus HCoV-229E S-protein structure and receptor binding. *eLife* **8**, e51230 (2019). doi:10.7554/eLife.51230 Medline

30. T. Starr, A. J. Greaney, W. Hannon, J. Bloom, jbloomlab/SARS-CoV-2-RBD_DMS_variants: published version. Zenodo (2022); doi:10.5281/zenodo.6657545.

31. A. E. Wentz, E. V. Shusta, A novel high-throughput screen reveals yeast genes that increase secretion of heterologous proteins. *Appl. Environ. Microbiol.* **73**, 1189–1198 (2007). doi:10.1128/AEM.02427-06 Medline

32. K. H. D. Crawford, J. D. Bloom, alignparse: A Python package for parsing complex features from high-throughput long-read sequencing. *J. Open Source Softw.* **4**, 1915 (2019). doi:10.21105/joss.01915 Medline

33. R. M. Adams, T. Mora, A. M. Walczak, J. B. Kinney, Measuring the sequence-affinity landscape of antibodies with massively parallel titration curves. *eLife* **5**, e23156 (2016). doi:10.7554/eLife.23156 Medline

34. N. Peterman, E. Levine, Sort-seq under the hood: Implications of design choices on large-scale characterization of sequence-function relations. *BMC Genomics* **17**, 206 (2016). doi:10.1186/s12864-016-2533-5 Medline

35. M. Delignette-Muller, C. Dutang, fitdistrplus: An R Package for Fitting Distributions. *J. Statist. Softw. Art.* **64**, 1–34 (2015).

36. M. B. Doud, O. Ashenberg, J. D. Bloom, Site-Specific Amino Acid Preferences Are Mostly Conserved in Two Closely Related Protein Homologs. *Mol. Biol. Evol.* **32**, 2944–2960 (2015). doi:10.1093/molbev/msv167 Medline

37. K. H. D. Crawford, R. Eguia, A. S. Dingens, A. N. Loes, K. D. Malone, C. R. Wolf, H. Y. Chu, M. A. Tortorici, D. Veesler, M. Murphy, D. Pettie, N. P. King, A. B. Balazs, J. D. Bloom, Protocol and Reagents for Pseudotyping Lentiviral Particles with SARS-CoV-2 Spike Protein for Neutralization Assays. *Viruses* **12**, 513 (2020). doi:10.3390/v12050513 Medline

38. N. Shukla, S. M. Roelle, V. G. Suzart, A. M. Bruchez, K. A. Matreyek, Mutants of human ACE2 differentially promote SARS-CoV and SARS-CoV-2 spike mediated infection. *PLOS Pathog.* **17**, e1009715 (2021). doi:10.1371/journal.ppat.1009715 Medline

39. W. Kabsch, XDS. *Acta Crystallogr. D Biol. Crystallogr.* **66**, 125–132 (2010). doi:10.1107/S0907444909047337 Medline

40. A. J. McCoy, R. W. Grosse-Kunstleve, P. D. Adams, M. D. Winn, L. C. Storoni, R. J. Read, Phaser crystallographic software. *J. Appl. Crystallogr.* **40**, 658–674 (2007). doi:10.1107/S0021889807021206 Medline

41. P. Emsley, B. Lohkamp, W. G. Scott, K. Cowtan, Features and development of Coot. *Acta Crystallogr. D Biol. Crystallogr.* **66**, 486–501 (2010). doi:10.1107/S0907444910007493 Medline

42. T. I. Croll, ISOLDE: A physically realistic environment for model building into low-resolution electron-density maps. *Acta Crystallogr. D Struct. Biol.* **74**, 519–530 (2018). doi:10.1107/S2059798318002425 Medline

43. G. N. Murshudov, P. Skubák, A. A. Lebedev, N. S. Pannu, R. A. Steiner, R. A. Nicholls, M. D. Winn, F. Long, A. A. Vagin, REFMAC5 for the refinement of macromolecular crystal structures. *Acta Crystallogr. D Biol. Crystallogr.* **67**, 355–367 (2011). doi:10.1107/S0907444911001314 Medline

44. D. Liebschner, P. V. Afonine, M. L. Baker, G. Bunkóczi, V. B. Chen, T. I. Croll, B. Hintze, L. W. Hung, S. Jain, A. J. McCoy, N. W. Moriarty, R. D. Oeffner, B. K. Poon, M. G. Prisant, R. J. Read, J. S. Richardson, D. C. Richardson, M. D. Sammito, O. V. Sobolev, D. H. Stockwell, T. C. Terwilliger, A. G. Urzhumtsev, L. L. Videau, C. J. Williams, P. D. Adams, Macromolecular structure determination using X-rays, neutrons and electrons: Recent developments in Phenix. *Acta Crystallogr. D Struct. Biol.* **75**, 861–877 (2019). doi:10.1107/S2059798319011471 Medline

45. E. C. Thomson, L. E. Rosen, J. G. Shepherd, R. Spreafico, A. da Silva Filipe, J. A. Wojcechowskyj, C. Davis, L. Piccoli, D. J. Pascall, J. Dillen, S. Lytras, N. Czudnochowski, R. Shah, M. Meury, N. Jesudason, A. De Marco, K. Li, J. Bassi, A. O'Toole, D. Pinto, R. M. Colquhoun, K. Culap, B. Jackson, F. Zatta, A. Rambaut, S.

Jaconi, V. B. Sreenu, J. Nix, I. Zhang, R. F. Jarrett, W. G. Glass, M. Beltramello, K. Nomikou, M. Pizzuto, L. Tong, E. Cameroni, T. I. Croll, N. Johnson, J. Di Iulio, A. Wickenhagen, A. Ceschi, A. M. Harbison, D. Mair, P. Ferrari, K. Smollett, F. Sallusto, S. Carmichael, C. Garzoni, J. Nichols, M. Galli, J. Hughes, A. Riva, A. Ho, M. Schiuma, M. G. Semple, P. J. M. Openshaw, E. Fadda, J. K. Baillie, J. D. Chodera, S. J. Rihn, S. J. Lycett, H. W. Virgin, A. Telenti, D. Corti, D. L. Robertson, G. Snell; ISARIC4C Investigators; COVID-19 Genomics UK (COG-UK) Consortium, Circulating SARS-CoV-2 spike N439K variants maintain fitness while evading antibody-mediated immunity. *Cell* **184**, 1171–1187.e20 (2021). doi:10.1016/j.cell.2021.01.037 Medline

46. D. A. Case, H. Metin Aktulga, K. Belfon, I. Ben-Shalom, S. R. Brozell, D. S. Cerutti, T. E. Cheatham III, V. W. D. Cruzeiro, T. A. Darden, R. E. Duke, G. Giambasu, M. K. Gilson, H. Gohlke, A. W. Goetz, R. Harris, S. Izadi, S. A. Izmailov, C. Jin, K. Kasavajhala, M. C. Kaymak, E. King, A. Kovalenko, T. Kurtzman, T. Lee, S. LeGrand, P. Li, C. Lin, J. Liu, T. Luchko, R. Luo, M. Machado, V. Man, M. Manathunga, K. M. Merz, Y. Miao, O. Mikhailovskii, G. Monard, H. Nguyen, K. A. O'Hearn, A. Onufriev, F. Pan, S. Pantano, R. Qi, A. Rahnamoun, D. R. Roe, A. Roitberg, C. Sagui, S. Schott-Verdugo, J. Shen, C. L. Simmerling, N. R. Skrynnikov, J. Smith, J. Swails, R. C. Walker, J. Wang, H. Wei, R. M. Wolf, X. Wu, Y. Xue, D. M. York, S. Zhao, P. A. Kollman, *Amber 2021* (University of California, San Francisco, 2021).

47. J. A. Maier, C. Martinez, K. Kasavajhala, L. Wickstrom, K. E. Hauser, C. Simmerling, Ff14SB: Improving the accuracy of protein side chain and backbone parameters from ff99SB. *J. Chem. Theory Comput.* **11**, 3696–3713 (2015). doi:10.1021/acs.jctc.5b00255 Medline

48. K. N. Kirschner, A. B. Yongye, S. M. Tschampel, J. González-Outeiriño, C. R. Daniels, B. L. Foley, R. J. Woods, GLYCAM06: A generalizable biomolecular force field. Carbohydrates. *J. Comput. Chem.* **29**, 622–655 (2008). doi:10.1002/jcc.20820 Medline

49. W. L. Jorgensen, J. Chandrasekhar, J. D. Madura, R. W. Impey, M. L. Klein, Comparison of simple potential functions for simulating liquid water. *J. Chem. Phys.* **79**, 926–935 (1983). doi:10.1063/1.445869

50. I. S. Joung, T. E. Cheatham 3rd, Determination of alkali and halide monovalent ion parameters for use in explicitly solvated biomolecular simulations. *J. Phys. Chem. B* **112**, 9020–9041 (2008). doi:10.1021/jp8001614 Medline

51. P. Li, L. F. Song, K. M. Merz Jr., Systematic parameterization of monovalent ions employing the nonbonded model. *J. Chem. Theory Comput.* **11**, 1645–1657 (2015). doi:10.1021/ct500918t Medline

52. K. Hauser, B. Essuman, Y. He, E. Coutsias, M. Garcia-Diaz, C. Simmerling, A human transcription factor in search mode. *Nucleic Acids Res.* **44**, 63–74 (2016). doi:10.1093/nar/gkv1091 Medline

53. D. R. Roe, T. E. Cheatham 3rd, Parallelization of CPPTRAJ enables large scale analysis of molecular dynamics trajectory data. *J. Comput. Chem.* **39**, 2110–2117 (2018). doi:10.1002/jcc.25382 Medline

54. W. Humphrey, A. Dalke, K. Schulten, VMD: Visual molecular dynamics. *J. Mol. Graph.* **14**, 33–38, 27–28 (1996). doi:10.1016/0263-7855(96)00018-5 Medline

55. P. Han, L. Li, S. Liu, Q. Wang, D. Zhang, Z. Xu, P. Han, X. Li, Q. Peng, C. Su, B. Huang, D. Li, R. Zhang, M. Tian, L. Fu, Y. Gao, X. Zhao, K. Liu, J. Qi, G. F. Gao, P. Wang, Receptor binding and complex structures of human ACE2 to spike RBD from omicron and delta SARS-CoV-2. *Cell* **185**, 630–640.e10 (2022). doi:10.1016/j.cell.2022.01.001 Medline

56. T. Zhou, Y. Tsybovsky, J. Gorman, M. Rapp, G. Cerutti, G.-Y. Chuang, P. S. Katsamba, J. M. Sampson, A. Schön, J. Bimela, J. C. Boyington, A. Nazzari, A. S. Olia, W. Shi, M. Sastry, T. Stephens, J. Stuckey, I.-T. Teng, P. Wang, S. Wang, B. Zhang, R. A. Friesner, D. D. Ho, J. R. Mascola, L. Shapiro, P. D. Kwong, Cryo-EM Structures of SARS-CoV-2 Spike without and with ACE2 Reveal a pH-Dependent Switch to Mediate Endosomal Positioning of Receptor-Binding Domains. *Cell Host Microbe* **28**, 867–879.e5 (2020). doi:10.1016/j.chom.2020.11.004 Medline

57. X. Zhu, D. Mannar, S. S. Srivastava, A. M. Berezuk, J.-P. Demers, J. W. Saville, K. Leopold, W. Li, D. S. Dimitrov, K. S. Tuttle, S. Zhou, S. Chittori, S. Subramaniam, Cryo-electron microscopy structures of the N501Y SARS-CoV-2 spike protein in complex with ACE2 and 2 potent neutralizing antibodies. *PLOS Biol.* **19**, e3001237 (2021). doi:10.1371/journal.pbio.3001237 Medline

58. Y. Wang, C. Xu, Y. Wang, Q. Hong, C. Zhang, Z. Li, S. Xu, Q. Zuo, C. Liu, Z. Huang, Y. Cong, Conformational dynamics of the Beta and Kappa SARS-CoV-2 spike proteins and their complexes with ACE2 receptor revealed by cryo-EM. *Nat. Commun.* **12**, 7345 (2021). doi:10.1038/s41467-021-27350-0 Medline

59. B. J. Grant, A. P. C. Rodrigues, K. M. ElSawy, J. A. McCammon, L. S. D. Caves, Bio3d: An R package for the comparative analysis of protein structures. *Bioinformatics* **22**, 2695–2696 (2006). doi:10.1093/bioinformatics/btl461 Medline

60. J. VanderPlas, B. Granger, J. Heer, D. Moritz, K. Wongsuphasawat, A. Satyanarayan, E. Lees, I. Timofeev, B. Welsh, S. Sievert, Altair: Interactive Statistical Visualizations for Python. *J. Open Source Softw.* **3**, 1057 (2018). doi:10.21105/joss.01057

61. T. N. Starr, A. J. Greaney, A. Addetia, W. W. Hannon, M. C. Choudhary, A. S. Dingens, J. Z. Li, J. D. Bloom, Prospective mapping of viral mutations that escape antibodies used to treat COVID-19. *Science* **371**, 850–854 (2021). doi:10.1126/science.abf9302 Medline

62. A. Addetia, Y.-J. Park, T. Starr, A. J. Greaney, K. R. Sprouse, J. E. Bowen, S. W. Tiles, W. C. Van Voorhis, J. D. Bloom, D. Corti, A. C. Walls, D. Veesler, Structural changes in the SARS-CoV-2 spike E406W mutant escaping a clinical monoclonal antibody cocktail. bioRxiv 477288 [Preprint] (2022); doi:10.1101/2022.01.21.477288.
